# Supplementary material for: An enzymatic continuous-flow reactor based on a pore-size matching nano- and isoporous block copolymer membrane
Source: Nat Commun. 2024 Apr 17;15:3308. doi: 10.1038/s41467-024-47007-y (PMC11024217; doi:10.1038/s41467-024-47007-y)

Supplementary Information

An Enzymatic Continuous-Flow Reactor Based on a Pore-size Matching Nano- and Isoporous Block Copolymer Membrane

Zhenzhen Zhang[1†], Liang Gao[2†], Alexander Boes[3], Barbara Bajer[1], Johanna Stotz[2], Lina Apitius[3], Felix Jakob[3], Erik S. Schneider[1], Evgeni Sperling[1], Martin Held[1], Thomas Emmler[1], Ulrich Schwaneberg[2,3*] and Volker Abetz[1,4*]

1) Helmholtz-Zentrum Hereon, Institute of Membrane Research, Max-Planck-Straße 1, 21502 Geesthacht, Germany

2) RWTH Aachen University, Institute of Biotechnology, Worringerweg 3, 52074 Aachen, Germany

3) DWI – Leibniz Institute for Interactive Materials, Forckenbeckstraße 50, 52056 Aachen, Germany

4) Universität Hamburg, Institute of Physical Chemistry, Martin-Luther-King-Platz 6, 20146 Hamburg, Germany

† These authors contributed equally

* Corresponding authors: u.schwaneberg@biotec.rwth-aachen.de, volker.abetz@hereon.de

Inventory of Supporting Information:

1. Supplementary Methods

2. Supplementary Figures

2.1. Preparation of YmPh-WT and YmPh-LCI (Figure 1)

2.2. Enzyme immobilization on isoporous BCP membranes (Figure 2-7)

2.3. Localization of immobilized YmPh-LCI within the isoporous BCP membrane (Figure 8-11)

2.4. Performance of continuous-flow membrane reactor (Figure 12-13)

2.4.1. Calculation of diffusion coefficient and residence time of substrate InsP6 (Figure 14-15)

2.4.2. Effect of flux on continuous-flow reaction (Figure 16)

2.4.3. Effect of substrate concentration on continuous-flow reaction (Figure 17)

2.4.4. Stability of the immobilized enzyme (Figure 18-20)

2.4.5. Enhanced performance by tailoring BCP membrane nanochannels (Figure 21-31)

2.4.6. Long-term catalytic performance of NaMeR PC91 (Figure 32)

2.5. Binding efficiency of other enzymes within BCP membrane of Pri57 (Figure 33)

2.6. Synthesis of PS-*b*-P4VP diblock copolymers (Figure 34)

3. Supplementary Tables (Table 1-14)

4. Supplementary Notes (4.1-4.9)

5. Supplementary References

1. Supplementary Methods

1.1. Materials

All chemicals are of analytical grade or higher purity and were purchased from Merck (Darmstadt, Germany), AppliChem (Darmstadt, Germany), and Carl Roth (Karlsruhe, Germany) if not otherwise specified. All materials were used as received without further purification unless described specifically. Styrene was purified over a column filled with aluminum oxide and subsequently distilled from di-*n*-butyl magnesium (Sigma-Aldrich, USA, 1.0 M solution in heptane). 4-Vinylpyridine (4VP) was distilled under reduced pressure after being treated twice with ethylaluminium dichloride (Sigma-Aldrich, USA, 1M in *n*-hexane). The solvent used for polymerization was tetrahydrofuran (THF), purified over sodium metal, and titrated with *sec*-butyl lithium (*s*-BuLi, Sigma-Aldrich, USA, 1.4 M solution in cyclohexane).

1.2. Preparation of YmPh-WT and YmPh-LCI

YmPh-WT and YmPh-17Helix-TEV-LCI (YmPh-LCI) gene constructs integrated in pALXtreme-5b plasmid were generated in-house and overexpressed in *E. coli* BL21-Gold (DE3) LacI^{Q1} [1,2]. In detail, pre-cultures were inoculated from glycerol stocks with a sterile pipette tip into cultivation tubes containing LB medium (5 mL; 10 g L^{-1} tryptone, 10 g L^{-1} NaCl, 5 g L^{-1} yeast extract) supplemented with ampicillin (100 µg mL^{-1}) and cultivated overnight (37 °C, 250 rpm). Main cultures were inoculated in 500 mL shaking-flasks containing TB medium (100 mL; 12 g L^{-1} tryptone, 24 g L^{-1} yeast extract, 5 g L^{-1} glycerol, 2.31 g L^{-1} KH_2PO_4, and 12.5 g L^{-1} mM K_2HPO_4, supplemented with ampicillin (100 µg mL^{-1}) with an OD_{600} of 0.05. Main cultures were grown (30 °C, 250 rpm) until an OD_{600} of 0.6-0.8, and protein expression was induced by adding 0.1 mM isopropyl β-D-1-thiogalactopyranoside (IPTG). After protein expression (18 °C, 200 rpm, 20 h), the cell pellet was harvested by centrifuging (Sorvall, ThermoFischer Scientific, Germany, 4 °C, 4000 × g, 20 min). The collected cell pellet

was suspended in 50 mL NaOAc buffer (25 mM, pH 5.5) and lysed by a French press. The lysates were centrifuged (4 °C, 11963 × g, 30 min) and the supernatant was collected and filtered with a 0.45 µm filter before purification.

YmPh-WT and YmPh-LCI were purified using cation-exchange chromatography (HiTrap® SP High Performance, Cytiva, United States; ÄKTAprime plus, Cytiva, United States)[1,3]. The purified enzyme was dialyzed against NaOAc buffer (200 mL, 25 mM, pH 5.5) for 20 h at 4 °C, and the dialysis buffer was changed 5-6 times. The concentration of desalted protein was detected at 280 nm using a UV spectrophotometer (Nanodrop™ 2000; Thermo Fisher Scientific; Version 1.6) applying extinction coefficients (calculated with Benchling software 2023, https://benchling.com, M^{-1} cm^{-1}) and molecular masses (kDa) of purified proteins (YmPh-WT: 47.3 kDa, 49890 M^{-1} cm^{-1}; YmPh-LCI: 55.1 kDa, 73840 M^{-1} cm^{-1}). The protein size was determined by dynamic light scattering (DLS). The purified proteins were stored at -20 °C for further use.

1.3. Determination of the maximum excitation and emission wavelength of 4-methylumbelliferyl phosphate and 4-methylumbelliferone

To determine the maximum excitation and emission wavelength of 4-methylumbelliferyl phosphate (4-MUP) and 4-methylumbelliferone (4-MU), 100 µL 4-MUP (0.5 mM) or saturated supernatant of 4-MU (0.5 mM) solution in 250 mM NaOAc buffer (pH 5.5, 1 mM $CaCl_2$, 0.01% Tween-20) were filled in black 96-well microtiter plates (MTP) (PS, F-bottom; Greiner Bio-One GmbH, Germany) for full length scanning from 250 nm to 550 nm (Supplementary Figure 2).

1.4. Influence of incubation time on enzyme immobilization efficiency to isoporous BCP membrane

The immobilization and activity measurements were performed as described in the main manuscript. Briefly, YmPh-LCI (100 µL, 4.29 µM) was loaded onto isoporous BCP membrane

discs (6 mm in diameter) and incubated (MTP shaker, 25 °C, 600 rpm) for 10, 60, or 1320 min. The relative maximum of reaction rate was calculated related to the enzyme activity under 10 min binding time. All experiments were performed in triplicate.

1.5. Reversed-phase high-performance liquid chromatography (RP-HPLC) measurement of products in flow reaction

Analysis of InsP3-InsP6 species was conducted using reversed-phase high-performance liquid chromatography (RP-HPLC). An HPLC system (Nexera X2, Shimadzu Deutschland GmbH, Duisburg, Germany; Labsolutions Version 5.54 SP2) equipped with autosampler SIL-20AC HT, HPLC pump LC-20AD, column oven CTO-20AC and refractive index detector RID-20A (Shimadzu Deutschland GmbH, Duisburg, Germany) was used for product separation and quantification. The analytical column was reversed-phase Ultrasep ES 100 RP18 (6 µm, 250 mm × 3 mm) (Dr. Maisch, Ammerbuch-Entringen, Germany). The mobile phase was 51.4% v/v MeOH (gradient grade, Honeywell International, Offenbach, Germany), 47.4% v/v ddH_2O, 0.2% v/v formic acid, and 1.0% v/v tetrabutylammonium hydroxide (40% v/v in water, Sigma-Aldrich Chemie 86854, Taufkirchen, Germany). The pH was adjusted to 3.7-3.8 by the addition of 18 M H_2SO_4 (HPLC grade, Sigma-Aldrich Chemie 339741, Taufkirchen, Germany) and the mobile phase was degassed for 15 min in an ultrasonication bath. The column temperature was set to 40 °C. After equilibration with the mobile phase, 20 µL of the sample was injected and inositol phosphates were detected using a refractive index detector. The initial flux of 0.3 mL min^{-1} is kept for 30 s. Afterwards, the flux was linearly increased to 0.5 mL min^{-1} within 24.5 min, as the final flux for analysis. The total analysis time was set to 30 min.

1.6. DLS determination of protein size

The protein (YmPh-WT and YmPh-LCI) samples (200 µL, 0.5 mg mL^{-1}) in NaOAc buffer (250 mM, pH 5.5) were filled into wells of an MTP (transparent, Greiner Bio-One International GmbH, Germany) and the hydrodynamic diameter was detected using dynamic light scattering

(DLS, DynaPro Plate Reader III, Wyatt Technology Corporation, USA; Wyatt DYNAMICS Version 4.0.1.5) at 25 °C.

Similarly, commercial Au nanoparticles (Mono-Sulfo-NHS-Nanogold®, 200 µL, 0.4 mg mL^{-1}), YmPh-LCI and Mono-Sulfo-NHS-Nanogold® conjugated YmPh-LCI_Au (200 µL, 0.5 mg mL^{-1}) were also characterized via DLS at 25 °C.

1.7. Diffusion coefficient measurement of InsP6

A standard pulse field gradient based on spin-echo sequence (PFG-NMR) allows measurements of the tracer diffusion coefficient if a series of NMR spectra is taken while the overall gradient strength (*G*) is progressively incremented[5,6]. The resulting signal intensity of the PFG spin echo experiment is given in the following equation. Here, the attenuation of the obtained echo intensity is dependent on the gradient strength.

$$I = I_0 \cdot \exp(-D \cdot (\gamma \cdot G \cdot \delta)^2 \cdot (\Delta - \frac{\delta}{3}) \cdot 10^4) \quad (1)$$

where I is the measured signal intensity and I_0 is the maximum signal intensity. D (m^2 s^{-1}) is the tracer diffusion coefficient, also known as translational diffusion coefficient. γ represents the gyromagnetic ratio. δ corresponds to the duration of the gradient pulse and Δ is the diffusion time which corresponds to the time between the onsets of subsequent gradient pulses. G is the gradient strength (G cm^{-1}).

The calculation of the desired tracer diffusion coefficient D is done by fitting the obtained PFG-NMR data with an exponential decay.

$$-\ln\left(\frac{I}{I_0}\right) = G^2 \cdot \gamma^2 \cdot \delta^2 \cdot (\Delta - \frac{\delta}{3}) \cdot 10^4 \quad (2)$$

The slope of this fitting curve is used to determine the diffusion coefficient D.

PFG-NMR measurements were implemented on a Bruker 500 MHz Avance III HD NMR spectrometer, equipped with a gradient unit (Bruker GERAT, Diff30 probe head). Temperature

control was performed using 4% methanol in methanol-d4 (Bruker standard sample). The gradient unit was calibrated using a known sample (doped water sample, Bruker, D = 1.91 m^2 s^{-1}) at 25 °C. 380 µL InsP6 solutions in NaOAc buffer (250 mM, pH 5.5) with a concentration of 0.38 mM, 38.59 mM, and 100 mM were placed into NMR tubes, respectively. The sample measurements were performed at 23°C. Before measurement, each sample was tempered in the NMR magnet for at least 30 min until it reached 23 °C. During the course of the experiment, the temperature was thoroughly checked. The temperature change of the sample during the repeated measurements was found to be at < 0.1 °C. A diffusion time of 20 ms and a gradient pulse of 1 ms duration were used for all measurements. The gradient field strength was varied from about 4 G cm^{-1} (or 120 G cm^{-1}) up to 250 G cm^{-1} (doped water sample up to 128 G cm^{-1}) in 16 steps. For each step (spectrum), 16 scans were acquired. Each measurement was repeated five to ten times. The evaluation of the measurement results was performed using the Bruker Dynamics Center software (version 2.7.3). The measured diffusion coefficient values were averaged over all measurements of the sample.

In general, 5-10% errors due to convection in the sample, eddy current effects, and non-linearity of the transmitters have to be taken into account for the measurements; for samples with high proton density, radiation damping also occurs.

1.8. YmPh-LCI binding affinity to dense P4VP and positively charged P4VP thin film surface by surface plasmon resonance (SPR) spectroscopy

Poly(4-vinyl pyridine) (P4VP, molecular weight ≈ 60 kg mol^{-1}, Sigma Aldrich, product number 472344) was freshly dissolved in methanol with a concentration of 0.5% (m/v) with shaking (25 °C, 1100 rpm, 30 min). To get the homogeneously polymer-coated SPR chips, commercial gold-coated SPR chip was gently fixed on the spin coater (Laurell, WS-650SZ-6NPP/LITE) under vacuum mode, and then the P4VP solution (80 µL) was continuously dropped onto the gold surface after turning on the spin coater (acceleration 800 rpm s^{-1}, 2000 rpm, 1 min). The

P4VP-coated SPR chips were further heated (70 °C, 15 min) to evaporate the rest organic solvent and then rinsed with ddH_2O. Finally, the SPR chips were dried by N_2 gas flow and then were stored for further use.

P4VP-coated SPR chips were post-modified via quaternization using 1,4-diiodobutane (DIB) to obtain the positively charged P4VP (PC-P4VP) coated SPR chips[7]. In detail, the quaternization was performed by placing the chips into a reactor (i.e., a desiccator) with a predetermined amount of liquid DIB. The desiccator was evacuated by a diaphragm vacuum pump (Vacuubrand, Wertheim, Germany) to facilitate the formation of the DIB vapour phase. Afterwards, the evacuated desiccator was kept at room temperature for a certain time. The resulting chips were taken out from the venting desiccator and transferred to a vacuum oven at room temperature for 2 days to remove unreacted DIB.

The binding affinity of YmPh-LCI on P4VP and PC-P4VP thin film surface was investigated by SPR spectroscopy using an MP-SPR Navi™ 210A VASA dual-channel SPR system (BioNavis Ltd, Tampere, Finland) at 784 nm[2]. In detail, YmPh-LCI solutions (250 µL, 0.04-1 µM) in Tris-HCl buffer (50 mM, pH 8.0) were flown over the P4VP- or PC-P4VP-coated SPR chip, while the position of the plasmons was recorded. After the injection of protein, Tris-HCl buffer (50 mM, pH 8.0) was continuously introduced. The amount of adsorbed YmPh-LCI was extracted from the sensor response in peak position (*deg*) and was calculated as the difference between the baseline in the buffer before and after protein injection (Δdeg), and converted into surface coverage in µg cm^{-2} (0.1 Δdeg = 0.1 µg cm^{-2}) using SPR-Navi Data Viewer (Version 6.7.0.9). To compare the surface coverage of YmPh-LCI on P4VP and PC-P4VP thin film surfaces, the saturated binding curve (0.5 µM protein) was integrated and analysed using the software TraceDrawer (Version 1.5).

1.9. Protein structure generation and dimension calculation

YmPh-LCI protein structure was generated using the AlphaFold2 software package on google's Alphafold server (https://github.com/sokrypton/ColabFold) with the amino acid sequence found in Supplementary Notes 4.1-4.3. Based on the obtained structure, the protein dimensions of YmPh-LCI were determined with PyMol suite (Version 2.5.8) by executing the script: Draw_Protein_Dimensions.py (https://pymolwiki.org/index.php/Draw_Protein_Dimensions).

1.10. GalOx$_{M3-5}$-LCI expression and purification

Galactose oxidase mutant M3-5 (GalOx$_{M3-5}$) exhibits a great sustainable route to produce building blocks of biobased polymers through biocatalytic oxidation of 5-Hydroxymethylfurfural (HMF)[8,9]. GalOx$_{M3-5}$-17Helix-TEV-LCI (GalOx$_{M3-5}$-LCI) gene construct integrated in pALXtreme-5b plasmid was generated in-house and overexpressed in *E. coli* BL21-Gold (DE3) LacI^{Q1}. In detail, pre-cultures were inoculated from glycerol stocks with a sterile pipette tip into cultivation tubes containing LB medium (5 mL; 10 g L^{-1} tryptone, 10 g L^{-1} NaCl, 5 g L^{-1} yeast extract) supplemented with ampicillin (100 µg mL^{-1}) and cultivated overnight (37 °C, 250 rpm). Main cultures were inoculated in 500 mL shaking-flasks containing TB medium (100 mL; 12 g L^{-1} tryptone, 24 g L^{-1} yeast extract, 5 g L^{-1} glycerol, 2.31 g L^{-1} KH_2PO_4, and 12.5 g L^{-1} mM K_2HPO_4, supplemented with ampicillin (100 µg mL^{-1}) with an OD_{600} of 0.05. When main cultures were grown (37 °C, 250 rpm) until an OD_{600} of 0.6-0.8, protein expression was induced by adding 0.1 mM isopropyl β-D-1-thiogalactopyranoside (IPTG) with 0.4 mM $CuSO_4$. After protein expression (25 °C, 200 rpm, 20 h), the cell pellet was harvested by centrifuging (Sorvall, ThermoFischer Scientific, Germany, 4 °C, 4000 × g, 20 min). The collected cell pellet was suspended in 40 mL Tris-HCI buffer (50 mM, pH 8.0) and lysed by a French press. The lysates were centrifuged (4 °C, 11963 × g, 30 min) and the supernatant was collected and filtered with a 0.45 µm filter before purification.

$GalOx_{M3-5}$-LCI was purified by using anion-exchange chromatography (HiTrap® Q HP column, Cytiva, United States; ÄKTAprime plus, Cytiva, United States). In detail, the filtered supernatant was subsequently loaded into the anion-exchange column (bed volume 5 mL, equilibrated with Tris-HCI buffer (50 mM, pH 8.0)), and $GalOx_{M3-5}$-LCI was then eluted under the NaCl gradient (1 M; 0% to 100% in 100 mL) in Tris-HCI buffer (50 mM, pH 8.0). The elution samples (2 mL/tube) were analysed with SDS-PAGE. Tubes of purified enzymes were collected in dialysis bag (14 kDa cut-off) and incubated in dialysis buffer (50 mM sodium phosphate buffer (NaPi), pH 7.0) with CuSO4 (0.4 mM) for 24 h at 4 °C. Afterwards, the collected samples were dialyzed against NaPi buffer (50 mM, pH 7.0; 400 mL) for 24 h at 4 °C and the dialysis buffer was changed 2-3 times. The concentration of desalted protein was detected at 280 nm using a UV spectrophotometer (Nanodrop™ 2000; Thermo Fisher Scientific; Version 1.6) applying extinction coefficients (calculated with Benchling software 2023, https://benchling.com, M^{-1} cm^{-1}) and molecular masses (kDa) of purified proteins ($GalOx_{M3-5}$-LCI: 76.4 kDa, 142585 M^{-1} cm^{-1}). The purified proteins were stored at -20 °C for further use.

1.11. $GalOx_{M3-5}$-LCI binding to isoporous membrane and the activity detection by ABTS assay

100 µL washing solution C (50 mM NaPi buffer, pH 7.0, 4 mM Triton X100) was applied to prewash the membrane (6 mm diameter) with pipetting up and down (membrane preparation see Methods section of the main manuscript). Afterwards, the prewashed membrane was washed again with 100 µL washing solution D (50 mM NaPi buffer, pH 7.0) twice. Subsequently, the membrane was washed with 100 µL binding solution (50 mM Tris-HCI buffer, pH 8.0) followed by 100 µL protein solution (ranging from 0-8.58 µM) diluted in the binding solution. After incubation on a MTP shaker (ELMI SkyLine DTS-4 Digital Thermo Shaker; Elminorthamerica Ltd.; 10 min, 600 rpm, 25 °C), the protein-loaded membrane was

washed with 100 µL washing solution C (MTP shaker, 5 min, 600 rpm, 25 °C). The washing steps were repeated three times. Finally, the washed membrane was pinched from MTP using tweezers and dried using nitrogen gas. Membrane pieces were transferred to a new transparent MTP (white, PS, Greiner bio-one GmbH) facing up before starting the ABTS (2,2'-azino-bis (3-ethylbenzothiazoline-6-sulfonic acid)) assay[10].

For determination of the enzymatic activity after immobilization on the membrane, 100 µL reaction solution containing ABTS (1 mM), HRP (0.005 g L^{-1}), and HMF (5 mM) in NaPi buffer (50 mM, pH 7.0) was loaded to each well of an MTP. The relative absorbance over time was measured under wavelength of 420 nm using a Tecan Sunrise microtiter plate reader (Tecan Trading AG, kinetic cycles: 400, kinetic interval: 3 s, room temperature; XFLUOR4 Version 4.51). The initial reaction rate was calculated to demonstrate the enzymatic activity of the catalytic membrane. All experiments were performed in triplicate.

1.12. *Candida antarctica* lipase B-LCI expression and purification

Candida antarctica lipase B (CaLB) is well-known as an industrially important enzyme[11]. CaLB-17Helix-TEV-LCI (CaLB-LCI) gene construct integrated in pGAPzαA plasmid was generated in-house and expressed in *Pichia pastoris* SMD1168[12]. *P. pastoris* cells containing CaLB-LCI plasmid were pre-grown on YPD agar plate (48 h, 30 °C; 10 g/L yeast extract, 20 g L^{-1} peptone, 20 g L^{-1} dextrose, 20 g L^{-1} agar, 100 µg mL^{-1} zeocin) and then a single colony was inoculated in YPD medium (16 h, 30 °C, 200 rpm, 10 mL; 10 g/L yeast extract, 20 g L^{-1} peptone, 20 g L^{-1} dextrose, 50 µg mL^{-1} zeocin) as a pre-culture. The main culture was prepared with an inoculation 0.4 mL pre-culture in 200 mL YPD medium in flask (1 L) and incubated under 30 °C, 200 rpm for 72 h. After main culture, cells were harvested (4 °C, 3113 × g, 30 min) and the supernatant containing secreted enzymes was collected. Tris-acetate buffer (250 mM, pH 7.0) was added in the supernatant with a volume ratio of 1:10 and then filtered with a 0.45 µm filter before purification.

The supernatant containing CaLB-LCI was concentrated (15 folds) and equilibrated slowly with ammonium acetate (0.8 M). CaLB-LCI was purified by using hydrophobic interaction chromatography (HIC) (packed volume 100 mL; Fractogel TSK Butyl 650 Size S, Merck, Darmstadt, Germany;) and ÄKTA system (GE Healthcare, Chalfont St Giles, UK). The column was equilibrated with Tris-acetate buffer (250 mL, 25 mM, pH 7.0). After loading the concentrated supernatant (flow rate 2 mL min^{-1}), the protein was eluted with a gradient elution from 100% Tris-acetate buffer (25 mM, pH 7.0) containing ammonium acetate (0.8 M) to 100% water (0%-100% in 100 mL) and collected in aliquots of 2 mL/tube. After SDS-APGE analysis of elution aliquots, purified CaLB-LCI samples were collected and buffer exchanged with triethanolamine buffer (TEA; 100 mM, pH 7.5). The concentration of purified protein was detected at 280 nm using a UV spectrophotometer (Nanodrop™ 2000; Thermo Fisher Scientific; Version 1.6) applying extinction coefficients (calculated with Benchling 2023 software, https://benchling.com, M^{-1} cm^{-1}) and molecular masses (kDa) of purified proteins (CaLB-LCI: 40.9 kDa, 65235 M^{-1} cm^{-1}). The purified proteins were stored at -20 °C for further use.

1.13. CaLB-LCI binding to isoporous membrane and the activity detection by *p*NPB assay

100 µL washing solution E (100 mM TEA buffer, pH 7.5, 0.1% (v/v) Tween 20) was applied to prewash the membrane (6 mm diameter) with pipetting up and down (membrane preparation see Methods section of the main manuscript). Afterwards, the prewashed membrane was washed again with 100 µL washing solution F (100 mM TEA buffer, pH 7.5) twice. Subsequently, the membrane was washed with 100 µL binding solution (50 mM Tris-HCI buffer, pH 8.0) followed by 100 µL protein solution (ranging from 0-21.45 µM) diluted in the binding solution. After incubation on a MTP shaker (ELMI SkyLine DTS-4 Digital Thermo Shaker; Elminorthamerica Ltd.; 10 min, 600 rpm, 25 °C), the protein-loaded membrane was

washed with 100 µL washing solution E (MTP shaker, 5 min, 600 rpm, 25 °C). The washing steps were repeated three times. Finally, the washed membrane was pinched from MTP using tweezers and dried using nitrogen gas. Membrane pieces were transferred to a new transparent MTP (white, PS, Greiner bio-one GmbH) facing up before starting the *p*NPB (*p*-Nitrophenyl Butyrate) assay.

For determination of the enzymatic activity after immobilization on the membrane, 100 µL reaction solution containing *p*NPB (0.5 mM, originally dissolved in acetonitrile) in TEA buffer (100 mM, pH 7.5) was loaded to each well of an MTP. The relative absorbance over time was measured under wavelength of 410 nm using a Tecan Sunrise microtiter plate reader (Tecan Trading AG, kinetic cycles: 100, kinetic interval: 12 s, room temperature; XFLUOR4 Version 4.51). The initial reaction rate was calculated to demonstrate the enzymatic activity of the catalytic membrane. All experiments were performed in triplicate.

2. Supplementary Figures

2.1. Preparation of YmPh-WT and YmPh-LCI

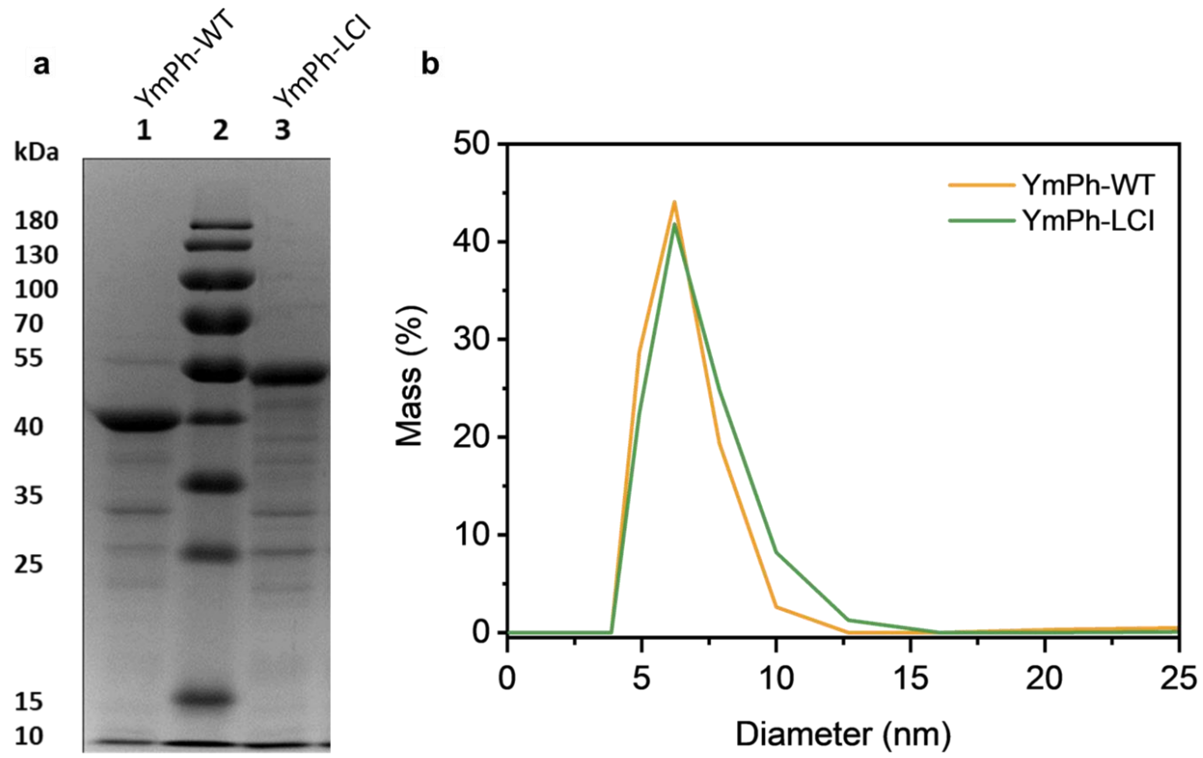

Supplementary Figure 1. Protein purification and size detection of YmPh-WT and YmPh-LCI. (a) Sodium dodecyl sulfate polyacrylamide gel electrophoresis (SDS-PAGE) analysis of purified protein (Image Lab Version 6.1). Lane 1: purified wild-type YmPh (YmPh-WT, MW = 47.3 kDa); Lane 2: PageRuler^{TM} Prestained Protein Ladder; Lane 3: purified YmPh-LCI (YmPh-LCI, MW = 55.1 kDa). (b) Dynamic light scattering (DLS) determination of protein size. Source data are provided as a Source Data file.

2.2. Enzyme immobilization on isoporous BCP membranes

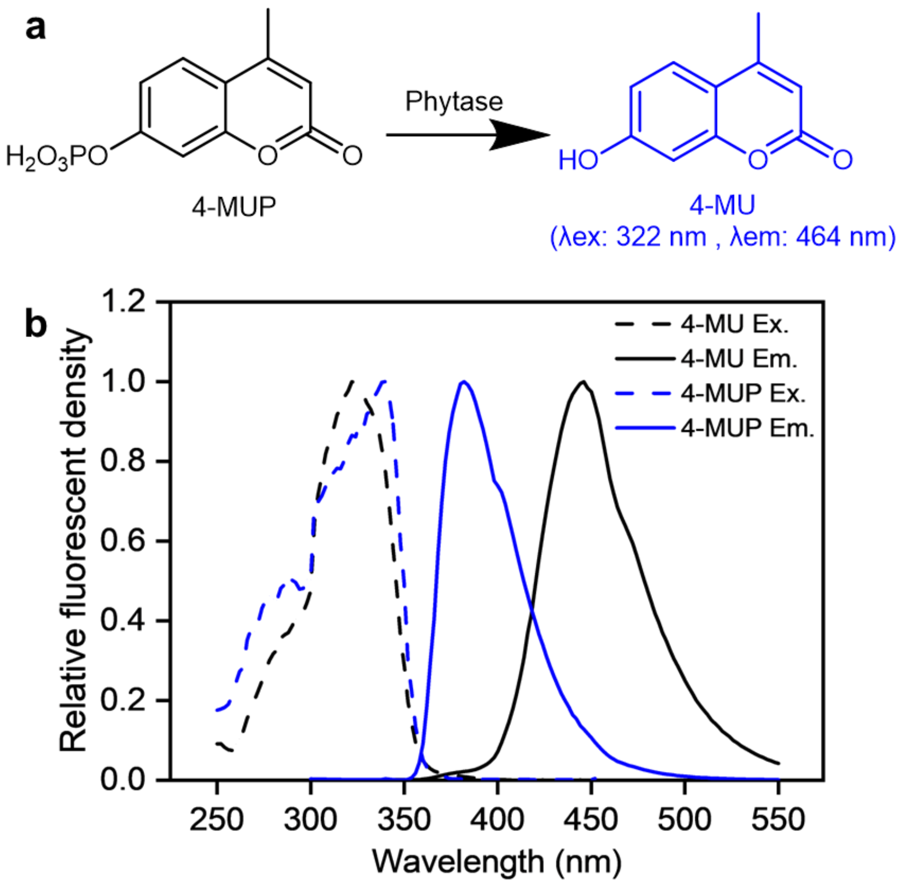

Supplementary Figure 2. 4-methylumbelliferyl phosphate (4-MUP) assay of phytase. (a) Enzymatic reaction scheme by using the 4-methylumbelliferyl phosphate (4-MUP) assay. (b) Wavelength scanning of substrate 4-MUP and product 4-methylumbelliferone (4-MU). The relative fluorescent intensity was measured using a Tecan Infinite® M1000 microtiter plate reader from 250 nm to 550 nm. The blue and black dashed lines represent the excitation wavelength (Ex.) of 4-MUP and 4-MU, respectively. The blue and black solid lines represent the emission wavelength (Em.) of 4-MUP and 4-MU, respectively. λ_{ex} = 322 nm and λ_{em} = 464 nm were used for the 4-MUP assay. Source data are provided as a Source Data file.

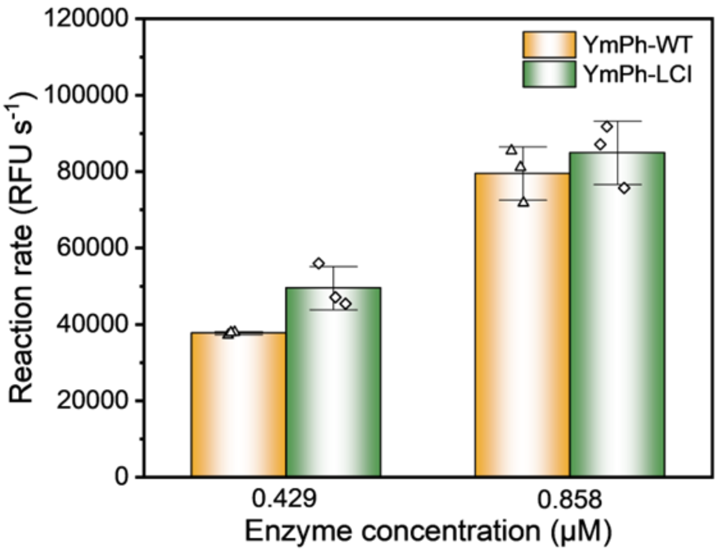

Supplementary Figure 3. Activity determination of purified YmPh-WT and YmPh-LCI using the 4-MUP assay. The initial reaction rate was calculated from two different protein concentrations (0.429 and 0.858 μM). Error bars represent s.d. of the mean from three independent experiments (n = 3). Source data are provided as a Source Data file.

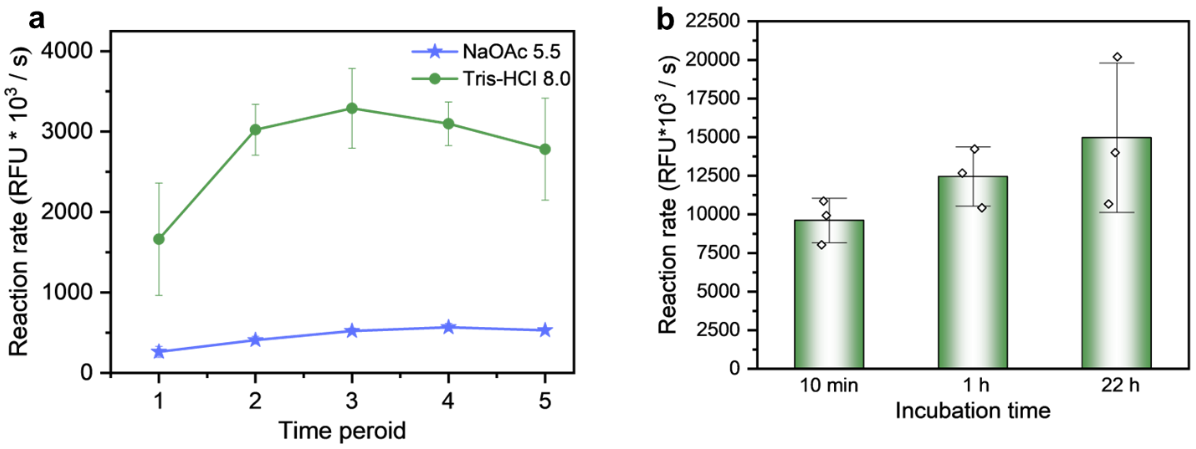

Supplementary Figure 4. Optimization of enzyme immobilization within BCP membranes. Effect of (a) buffer (at 0.858 µM enzyme) and (b) incubation time (at 4.29 µM enzyme) on the binding behaviour of YmPh-LCI on isoporous BCP membrane. The reaction rate of each time period was calculated as 300 seconds. Time period 1: 0.5-5.5 min; Time period 2: 5.5-10.5 min; Time period 3: 10.5-15.5 min; Time period 4: 15.5-20.5 min; Time period 5: 20.5-25.5 min. All error bars represent s.d. of the mean from three independent experiments (n = 3). Source data are provided as a Source Data file.

To probe the binding properties of enzymes on isoporous BCP membranes, we investigated different buffer solutions and varied the incubation time (Supplementary Figure 4). Tris-HCl buffer (50 mM, pH 8.0) for immobilization significantly improves the reaction rate when compared to NaOAc buffer (25 mM, pH 5.5), indicating that Tris-HCl buffer (50 mM, pH 8.0) improves enzyme binding to isoporous BCP membranes. When the incubation time was increased from 10 min to 22 h, no significant change of activity was detected indicating that the isoporous BCP membrane saturation takes place within the first 10 min. The optimum condition for YmPh-LCI is Tris-HCl buffer (50 mM, pH 8.0) and an incubation time of 10 min.

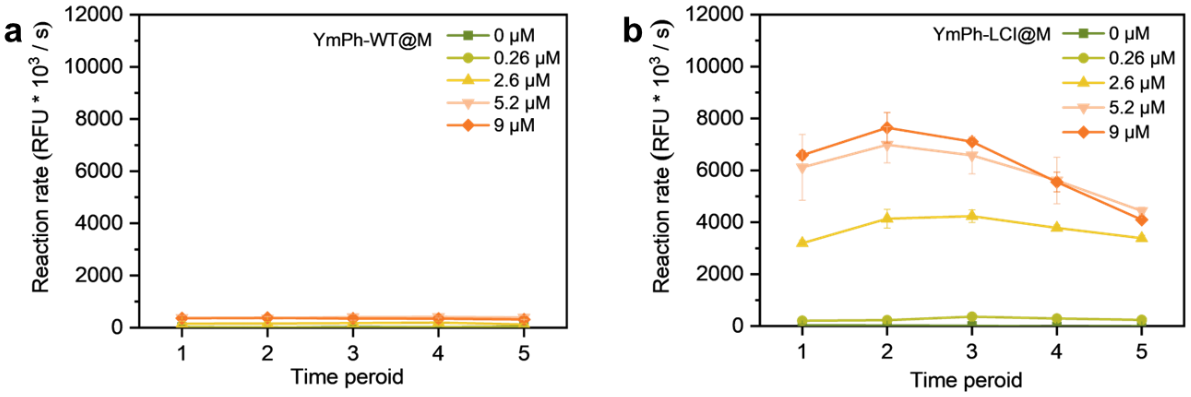

Supplementary Figure 5. Reaction rate of immobilized enzymes within BCP membranes. Effect of enzyme concentration on reaction rate for YmPh-WT and YmPh-LCI after immobilization on isoporous BCP membranes over time, determined by the 4-MUP assay using (a) YmPh-WT and (b) YmPh-LCI (both 0-9 μM). The relative fluorescence over time (0.5, 5.5, 10.5, 15.5, 20.5, 25.5 min) was measured using a Tecan Infinite® M1000 microtiter plate reader (λ_{ex}: 322 nm, λ_{em}: 464 nm; gain: 100, room temperature). The reaction rate of each time period was calculated as 300 seconds. Time period 1: 0.5-5.5 min; Time period 2: 5.5-10.5 min; Time period 3: 10.5-15.5 min; Time period 4: 15.5-20.5min; Time period 5: 20.5-25.5 min. All error bars represent s.d. of the mean from three independent experiments (n = 3). Source data are provided as a Source Data file.

Supplementary Figure 5 shows that the maximum reaction rate appeared in the time period 2 of 5.5-10.5 min instead of the time period 1 of 0.5-5.5 min. This is because the mass transport of substrate to the membrane nanoporous structure is limited and needs a certain time. Therefore, we used the maximum reaction rate of time period 2 (5.5-10.5 min) to evaluate the influence of enzyme concentration.

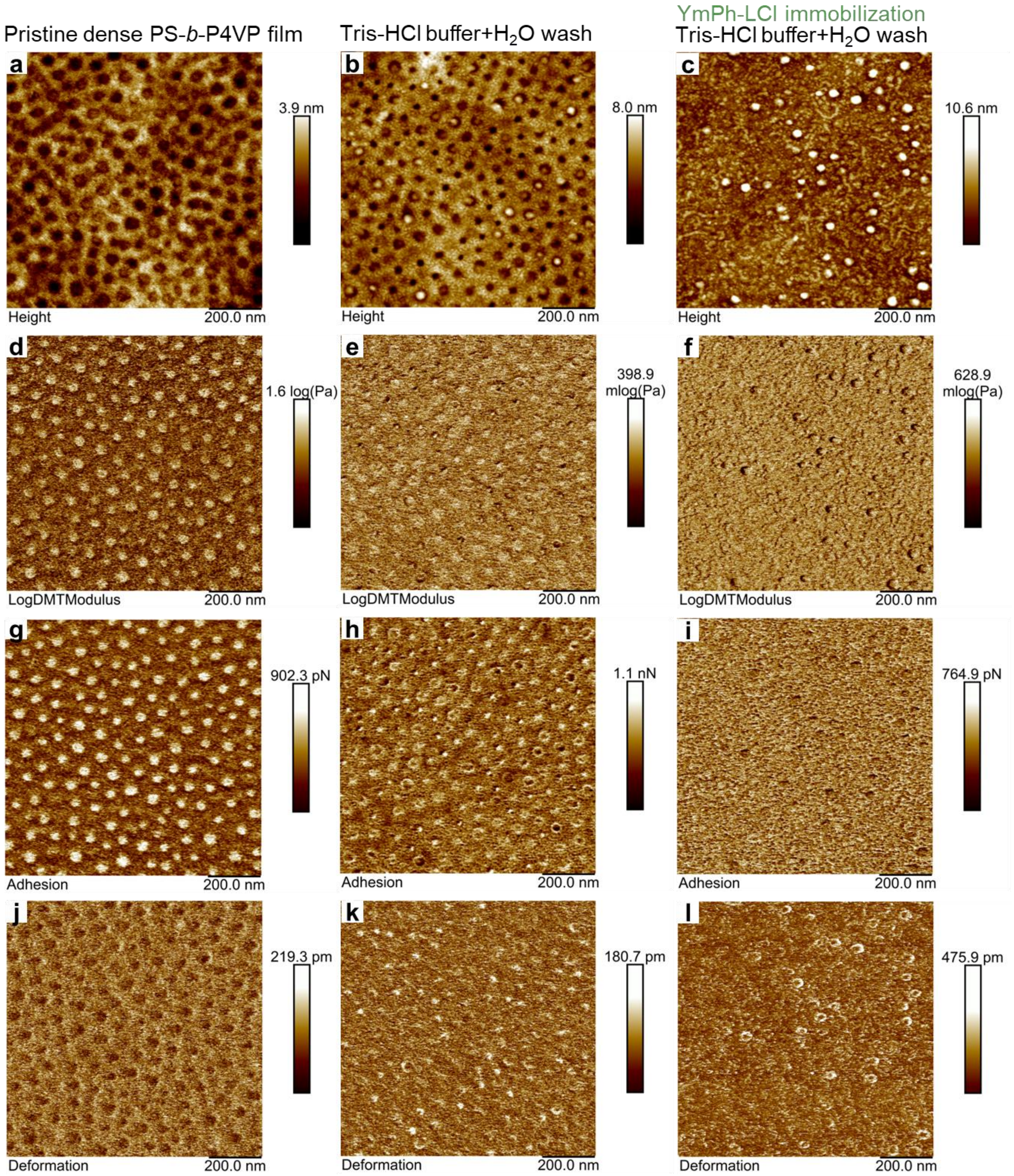

Supplementary Figure 6. Binding properties of YmPh-LCI on dense PS-*b*-P4VP films. AFM height, modulus, adhesion, and deformation images of (a, d, g, j) pristine dense PS-*b*-P4VP film, (b, e, h, k) after Tris-HCl buffer + H_2O wash and (c, f, i, l) after YmPh-LCI immobilization and subsequent Tris-HCl buffer + H_2O wash. The size of the image is 1 μm. Results were reproduced three times independently; representative micrographs are shown.

To investigate the YmPh-LCI binding behaviour onto a PS-*b*-P4VP substrate, we prepared dense PS-*b*-P4VP thin films on silicon wafers by spin-coating, and immobilized YmPh-LCI onto this film using the optimized incubation conditions with an excess amount of enzyme solution (400 µL, 5.2 µM YmPh-LCI). This amount of enzyme was enough to saturate 1 cm × 1 cm sized, flat thin film pieces. We measured the film topography and thickness by atomic force microscopy (AFM) in PeakForce QNM (Quantitative Nanomechanical Mapping) mode. The pristine dense film displays a well-defined microphase-separated structure with discrete P4VP domains in the continuous PS matrix based on its height, modulus, adhesion, and deformation maps (Supplementary Figure 6a, d, g, j). After treatment with only Tris-HCl buffer + H_2O wash, some bright domains appear in the height map (Supplementary Figure 6b), attributed to the swelling of P4VP domains under aqueous conditions. These swollen P4VP domains also appear in the height map of the film after YmPh-LCI immobilization (Supplementary Figure 6c). Interestingly, the contrast of nanomechanical properties in terms of elasticity, adhesion, and deformation between P4VP domains and the PS matrix decreases compared to pristine film, especially in the deformation map (Supplementary Figure 6d, g, j, e, h, k). In the YmPh-LCI immobilized film, the microphase-separated structure and the elasticity-, adhesion- and deformation-contrast of PS-*b*-P4VP disappear, whereas the new features like worms and nanodomains appear in the height map (Supplementary Figure 6c, f, i, l). Furthermore, we analyzed their roughness from height maps and compared them in Supplementary Table 3. YmPh-LCI treated films possessed a higher roughness than pristine films. Therefore, these results indicate that the immobilized YmPh-LCI formed a homogenous layer on top of the PS-*b*-P4VP films.

Additionally, we prepared scratched films to expose the blank silicon surface for the measurement of the film thickness. We determined the height difference between the film's surface and the silicon wafer by analyzing the corresponding AFM images (Supplementary

Figure 7). The thickness of the pristine dense film was 56.5 ± 2.1 nm, while the thickness of the YmPh-LCI treated film was 63.6 ± 0.9 nm. Therefore, the thickness of the immobilized YmPh-LCI layer was about 7.1 nm, corresponding to the size of YmPh-LCI. This indicates that YmPh-LCI forms a monolayer without layer-by-layer adsorptions or aggregates, which is beneficial to retain the native conformation and activity of the enzyme.

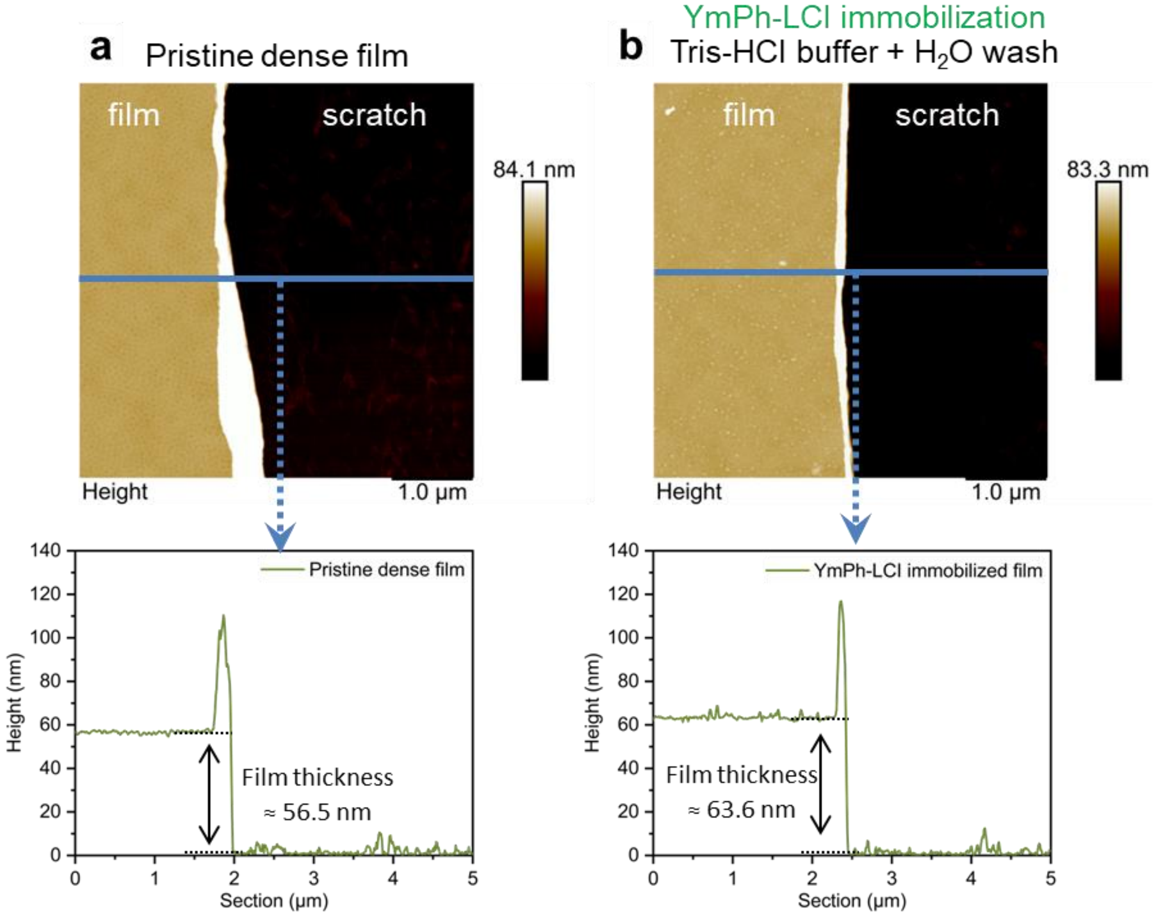

Supplementary Figure 7. Determination of the thickness of YmPh-LCI layer bound on dense PS-*b*-P4VP films. AFM height maps and the representative film thickness measurement of a (a) pristine dense PS-*b*-P4VP film, (b) after YmPh-LCI immobilization and subsequent Tris-HCl buffer + H_2O wash. The size of the images is 5 μm. Results were reproduced three times independently; representative micrographs are shown. Source data are provided as a Source Data file.

2.3. Localization of immobilized YmPh-LCI within the isoporous BCP membrane

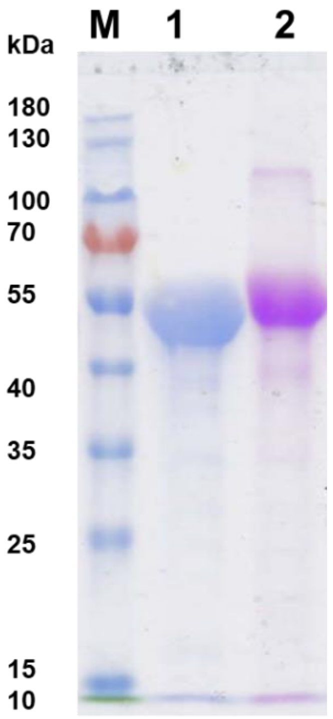

Supplementary Figure 8. Determination of the fluorescent labelling of enzyme by SDS-PAGE. M: PageRuler^{TM} Prestained Protein Ladder; Lane 1: Untreated YmPh-LCI; Lane 2: Cy3 conjugated YmPh-LCI (YmPh-LCI_Cy3). The size of Cy3 conjugated enzyme is larger than that of the untreated enzyme, indicating the success of the fluorescent labelling of enzyme. Source data are provided as a Source Data file.

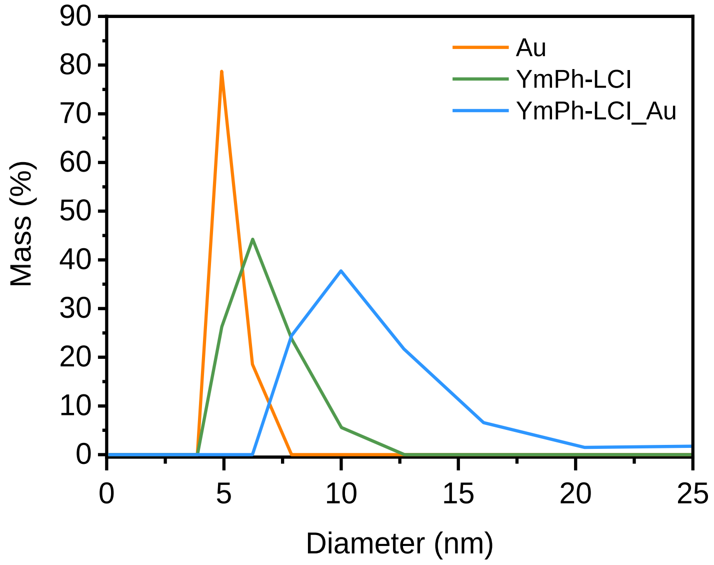

Supplementary Figure 9. Determination of gold nanoparticles modification of enzymes. Dynamic light scattering (DLS) measurements of Au nanoparticles (Mono-Sulfo-NHS-Nanogold®), YmPh-LCI, Mono-Sulfo-NHS-Nanogold® labeled YmPh-LCI (YmPh-LCI_Au). Source data are provided as a Source Data file.

Compared to Mono-Sulfo-NHS-Nanogold® and YmPh-LCI, Mono-Sulfo-NHS-Nanogold®-labelled YmPh-LCI (YmPh-LCI_Au) shows a clear shift in size, and no obvious signal in the size range of the Mono-Sulfo-NHS-Nanogold® indicating successful and complete conjugation between Mono-Sulfo-NHS-Nanogold® and YmPh-LCI.

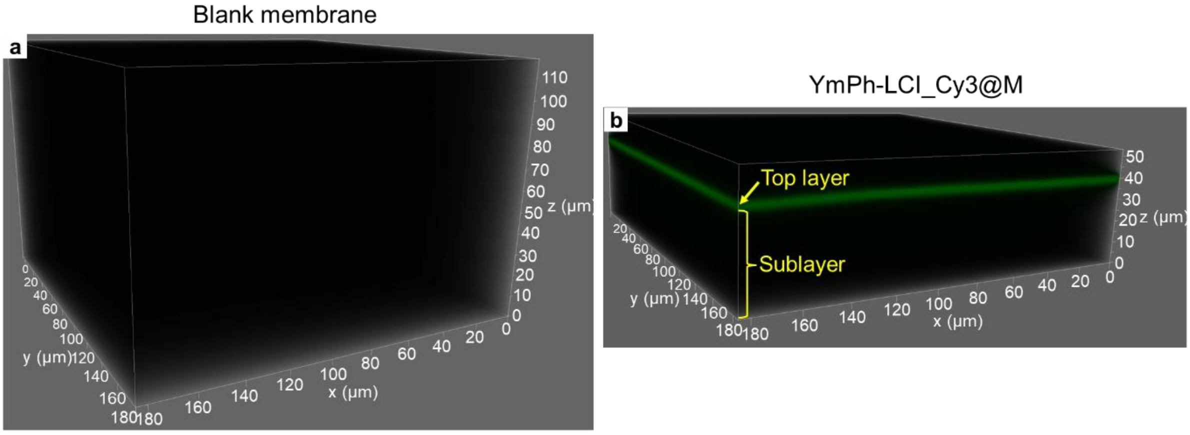

Supplementary Figure 10. Investigation of enzyme localization with BCP membranes by fluorescently labeled enzyme. Confocal 3D reconstruction images from a z-stack along the cross-section of membranes: (a) blank membrane without immobilized enzyme, (b) the membrane with the immobilized YmPh-LCI_Cy3 (YmPh-LCI_Cy3@M). The green fluorescence results from the immobilized YmPh-LCI_Cy3. The top surface of the membrane faces up. Results were reproduced three times independently; representative micrographs are shown. Source data are provided as a Source Data file.

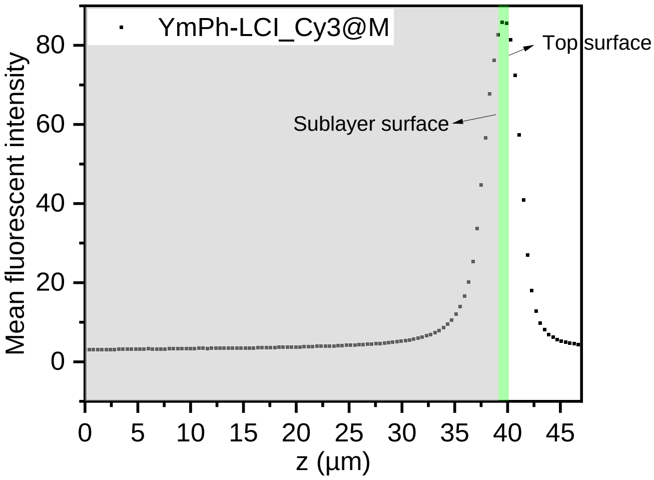

Supplementary Figure 11. Fluorescent intensity profile from a z-stack along the cross-section of the membrane. The top surface of the membrane is not completely flat, leading to a broader intensity profile than the ideal case. Source data are provided as a Source Data file.

For the blank membrane without the immobilized enzyme, there is no fluorescent background from the PS-*b*-P4VP membrane layer and non-woven support layer at the excitation wavelength of 552 nm (Supplementary Figure 10a). YmPh-LCI_Cy3 exhibits a concentrated

fluorescent signal from the top layer up to approx. 6 μm, where the strongest fluorescent signal is from the top layer of approx. 1 μm (Supplementary Figure 10b, Supplementary Figure 11). Therefore, the immobilized YmPh-LCI is predominantly located at the top layer rather than the macroporous sublayer.

2.4. Performance of continuous-flow membrane reactor

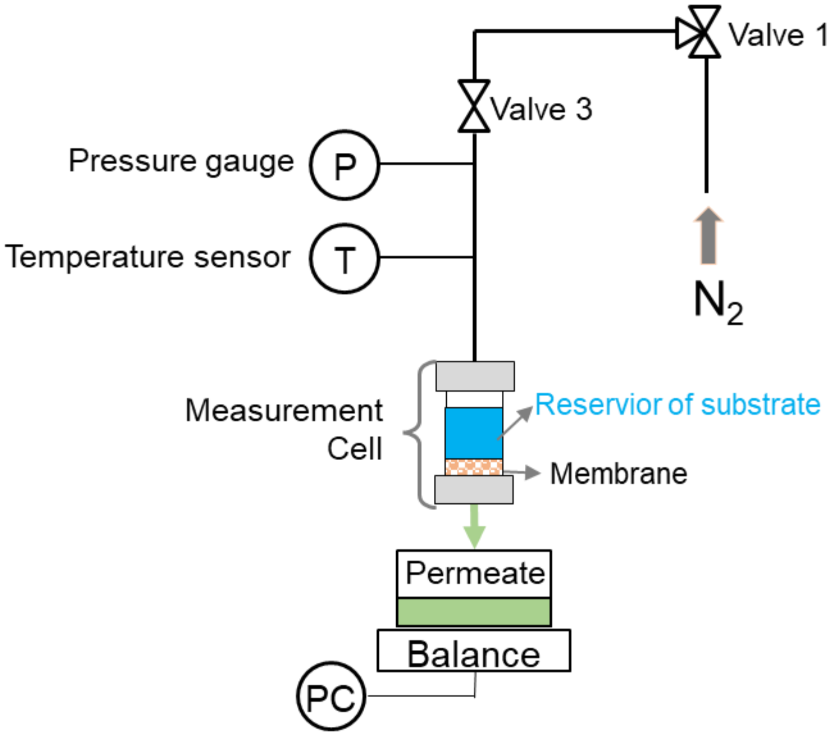

Supplementary Figure 12. Schematic representation of the pressure-driven filtration setup for the enzymatic reaction. The pressure-driven force is provided by the pressurized gas and controlled by pressure regulators (valves as shown in the scheme). Adapted with permission from ref [14]. Copyright 2018 John Wiley and Sons.

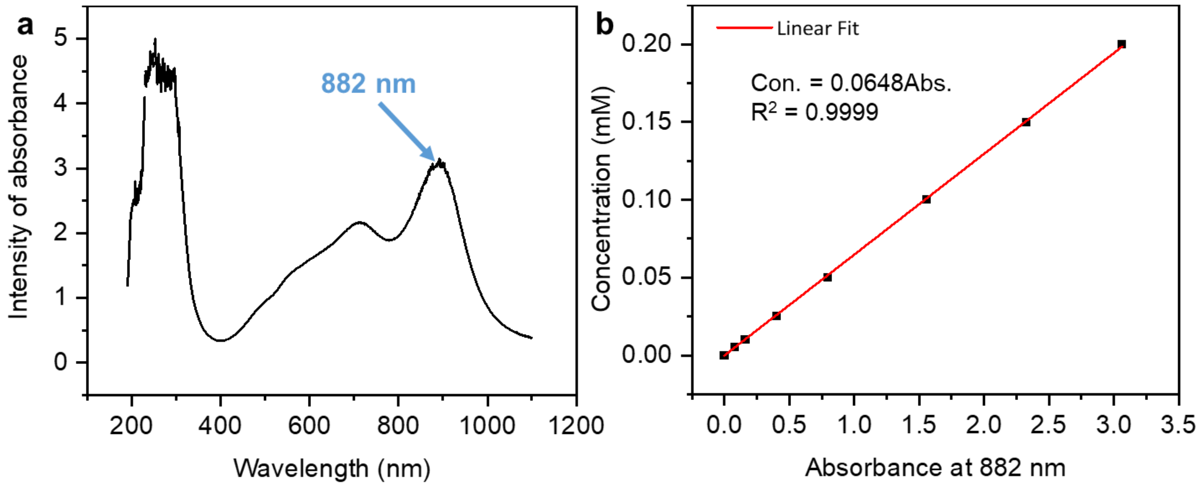

Supplementary Figure 13. Colorimetric assay for phosphate product detection. (a) UV-vis spectrum of the blue complex formed by colorimetric assay. (b) A standard calibration curve of aqueous KH_2PO_4 with a concentration range of 0-200 μM for the determination of phosphate concentration. Source data are provided as a Source Data file.

2.4.1. Calculation of diffusion coefficient and residence time of substrate InsP6

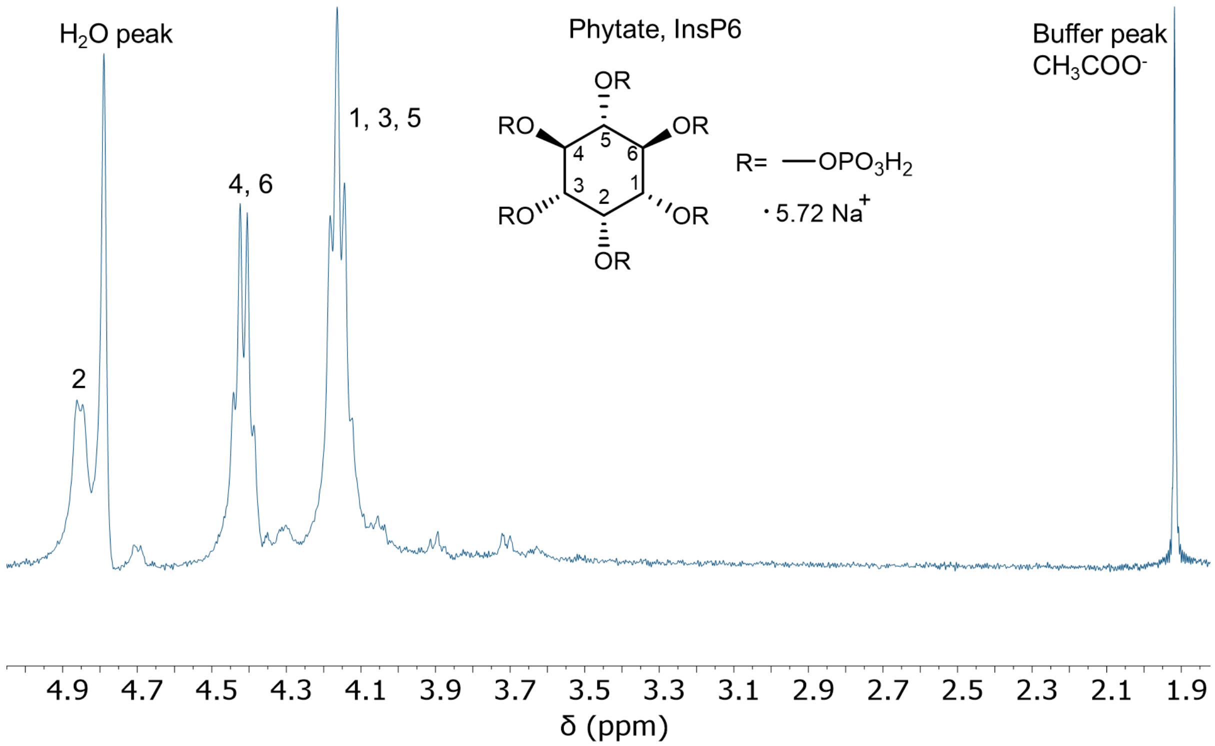

Supplementary Figure 14. The ^{1}H NMR spectrum of substrate phytate (InsP6). ^{1}H NMR (500 MHz, THF) δ 4.92 (s, 1H), 4.41 (q, 2H), 4.15 (q, 3H).

The hydraulic residence time corresponds to the residence time of substrate InsP6 inside the membrane without adsorptive interaction between InsP6 and membrane[15]. In our system, the majority of YmPh-LCI was immobilized in the isoporous, cylindrical top layer (as shown in Figure 2f-i of the main manuscript) and plays the dominant role in the enzymatic reaction due to its nanoconfinement effect. Therefore, we assume that the contribution of the macroporous sublayer to the residence time of InsP6 is negligible, and the hydraulic residence time within the isoporous, cylindrical top layer corresponds to the actual residence time of InsP6 (t) for the reaction.

The residence time inside the nanochannels of the cylindrical top layer can be calculated using the equation[15-17]:

$$t = \frac{V}{Q_{\mathrm{p}}} = \frac{A\ \cdot L\ \cdot \varepsilon}{A\ \cdot J_{\mathrm{p}}} \quad (3)$$

where V is volume of the nanochannels of the cylindrical top layer, Q_{p} is the flow rate, A is the membrane area, L is the thickness of the cylindrical top layer, and ε is the membrane porosity, measured with the software IMS (Imagic Bildverarbeitung AG, Opfikon, Switzerland) on the basis of the SEM images of membrane top surface and cross-section, J_{p} is the permeate flux. The corresponding data is shown in Supplementary Table 5.

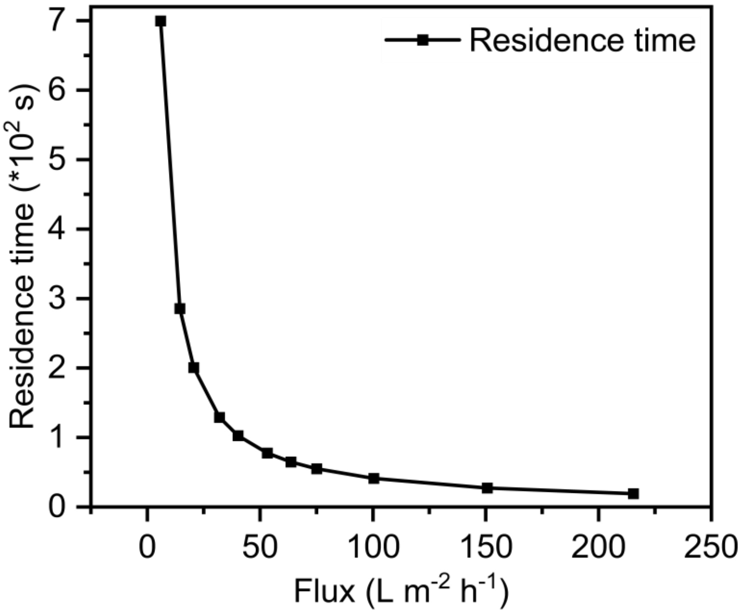

Supplementary Figure 15. Residence time of InsP6 inside the cylindrical top layer with varying flux. Source data are provided as a Source Data file.

The residence time of InsP6 inside the cylindrical top layer varies from 0.2×10^{-2} to 7.0×10^{-2} s for a flux decreasing from 215 to 6 L m^{-2} h^{-1}. The calculated radial diffusion time of InsP6 from the pore center to the pore wall is in the range of 2.41-2.72 $\times$ 10^{-6} (the details see Supplementary Table 5), which is about 4 orders of magnitude shorter than the residence time within the whole flux range. Therefore, the limitation of molecular diffusion within such narrow nanochannels is negligible.

2.4.2. Effect of flux on continuous-flow reaction

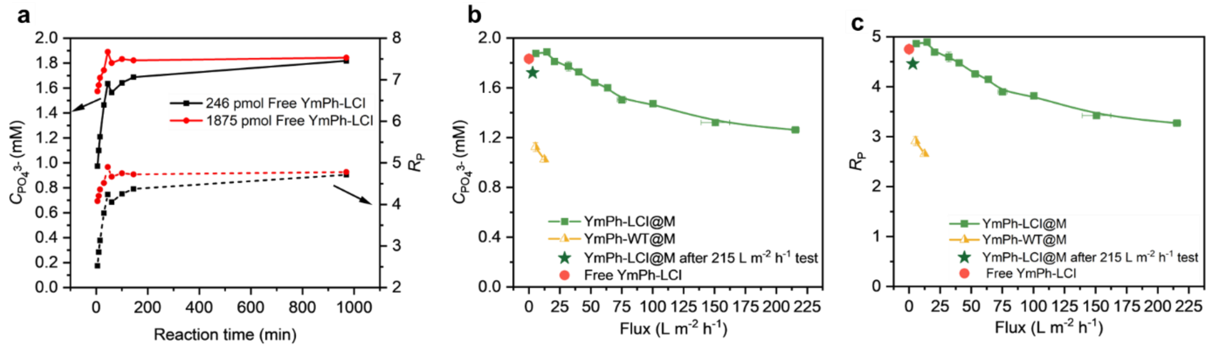

Supplementary Figure 16. Effect of protein concentration on the catalytic performance of free enzymes and effect of flux on that of immobilized enzymes. (a) Change of $C_{PO_4}{}^{3-}$ over reaction time from free YmPh-LCI in batch reaction with 10 mL 0.38 mM InsP6 at shaking with 90 rpm. Comparison of (b) $C_{PO_4}{}^{3-}$ and (c) R_P among free YmPh-LCI in batch reaction (1875 pmol, 16 h reaction, room temperature (21-23 ^{o}C)), YmPh-WT@M and YmPh-LCI@M in continuous-flow reaction with different fluxes. Substrate InsP6 concentration C_{InsP6} = 0.38 mM. YmPh-LCI@M shows a higher catalytic performance than YmPh-WT@M. All error bars represent s.d. of the mean from three independent experiments (n = 3). Source data are provided as a Source Data file.

To investigate the effect of free YmPh-LCI concentration on catalytic performance, we employed the different amounts of free YmPh-LCI (i.e., 246 pmol and 1875 pmol) incubated with InsP6 solution of C_{InsP6} = 0.38 mM, respectively (Supplementary Figure 16a). $C_{PO_4}{}^{3-}$ and R_P increase rapidly over reaction time up to 145 min for 246 pmol and 45 min for 1875 pmol, respectively, and afterwards level off until 16 h. Regardless of the amount of free YmPh-LCI, the maximum $C_{PO_4}{}^{3-}$ and R_P are comparable, $C_{PO_4}{}^{3-}$ = 1.82-1.83 mM and R_P = 4.7-4.8. It demonstrates that the amount of free YmPh-LCI barely affects the maximum catalytic performance.

2.4.3. Effect of substrate concentration on continuous-flow reaction

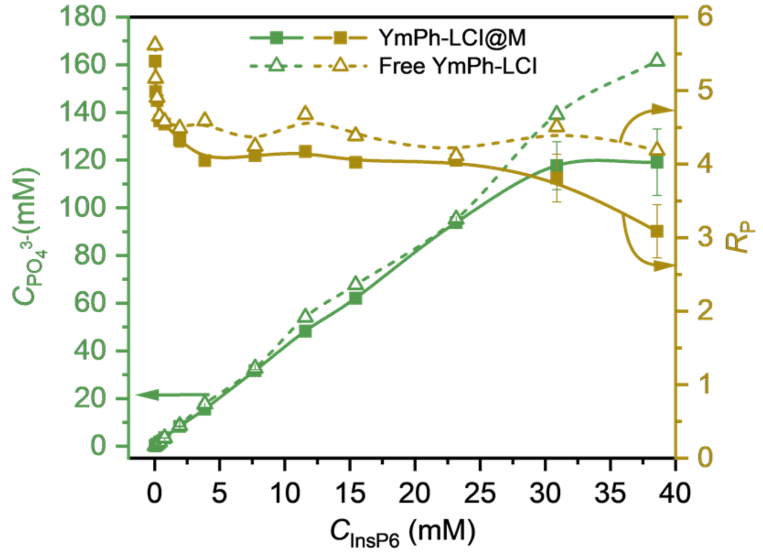

Supplementary Figure 17. Effect of C_{InsP6} on the catalytic performance (i.e., $C_{PO_4^{3-}}$ and R_P) of YmPh-LCI@M under optimal flux (~ 15 L m^{-2} h^{-1}) and free YmPh-LCI in batch reaction (enzyme amount 1000 pmol, 18 h reaction, room temperature (21-23 °C)). Error bars represent s.d. of the mean from three independent experiments (n = 3). Source data are provided as a Source Data file.

2.4.4. Stability of the immobilized enzyme

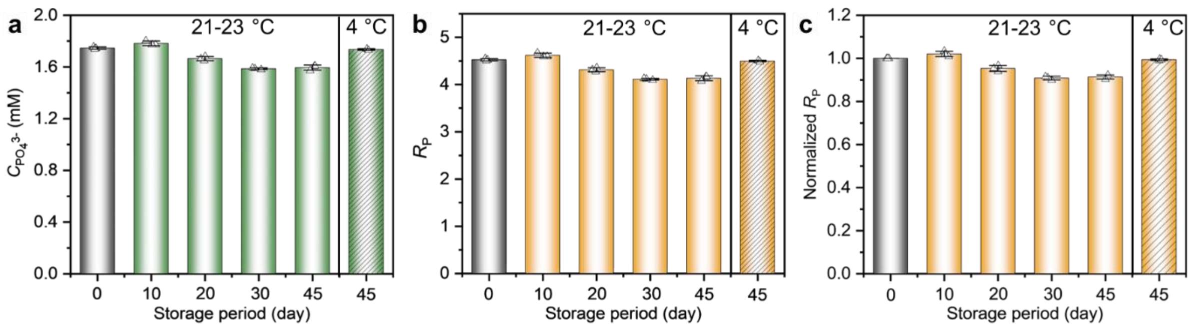

Supplementary Figure 18. Storage stability of YmPh-LCI@M. Change on (a) $C_{PO_4^{3-}}$, (b) R_P, and normalized R_P (c) with storage period at NaOAc buffer (25 mM, pH 5.5), room temperature (21-23 °C), or 4 °C. The activity decreases slowly to 90% over 30 days at room temperature, levelling off at 90% afterwards. All error bars represent s.d. of the mean from three independent experiments (n = 3). Source data are provided as a Source Data file.

Remarkably, YmPh-LCI@M exhibits superior storage stability preserving its activity up to 90% at room temperature (21-23 ºC) and 100% at 4 ºC after 45 days of storage (Supplementary Figure 18).

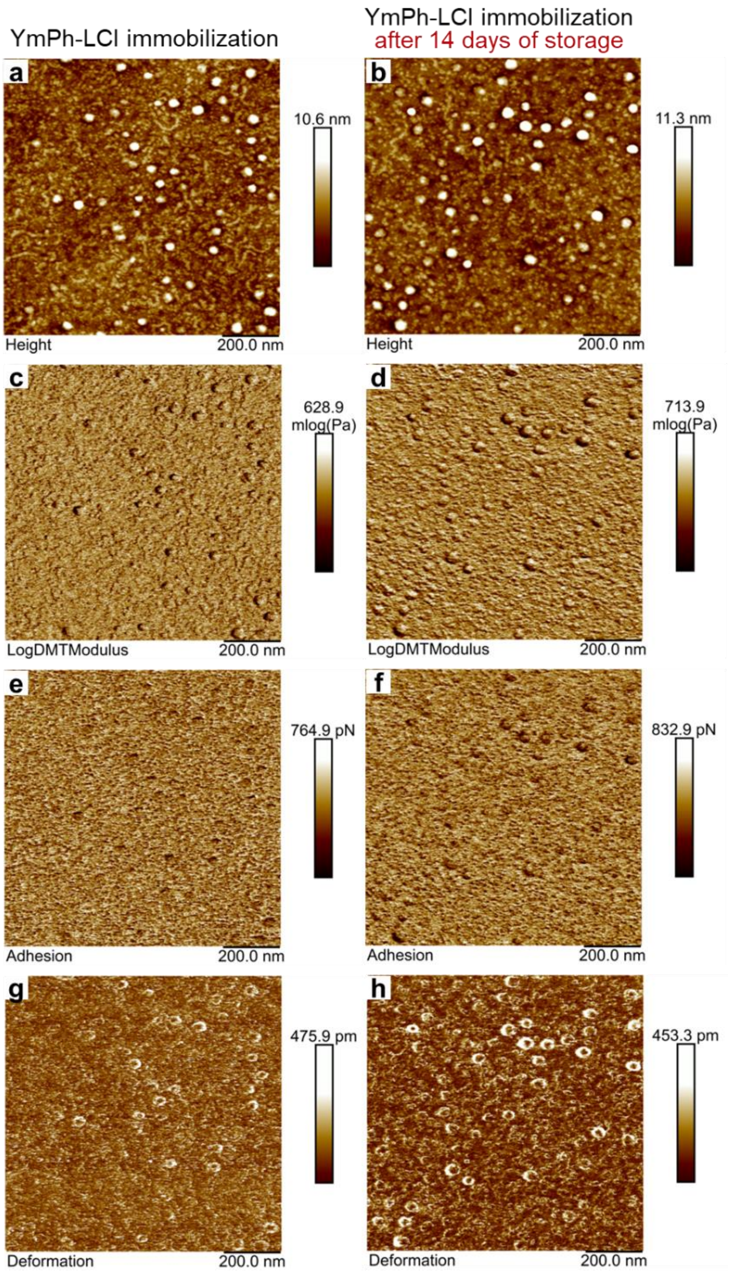

Supplementary Figure 19. AFM height, modulus, adhesion, and deformation images of PS-*b*-P4VP films after YmPh-LCI immobilization: (a, c, e, g) at day 0, (b, d, f, h) after 14 days of storage in NaOAc buffer (25 mM, pH 5.5) at room temperature (21-23 ºC). The size of the image is 1 µm. Results were reproduced three times independently; representative micrographs are shown.

To further confirm that the immobilized YmPh-LCI does not detach during storage, we analyzed the change of morphology of YmPh-LCI immobilized PS-*b*-P4VP dense film after 14 days of storage in NaOAc buffer (25 mM, pH 5.5) at room temperature (21-23 ^{o}C) (Supplementary Figure 19). We observed a similar morphology with a homogenous layer of immobilized enzyme and comparable roughness (i.e., $R_q \approx 1.07$ nm and $R_a \approx 0.84$ nm) when compared to reference measured at day 0 (Supplementary Figure 19a, b, Supplementary Figure 6c, f, i, l, and Supplementary Table 3).

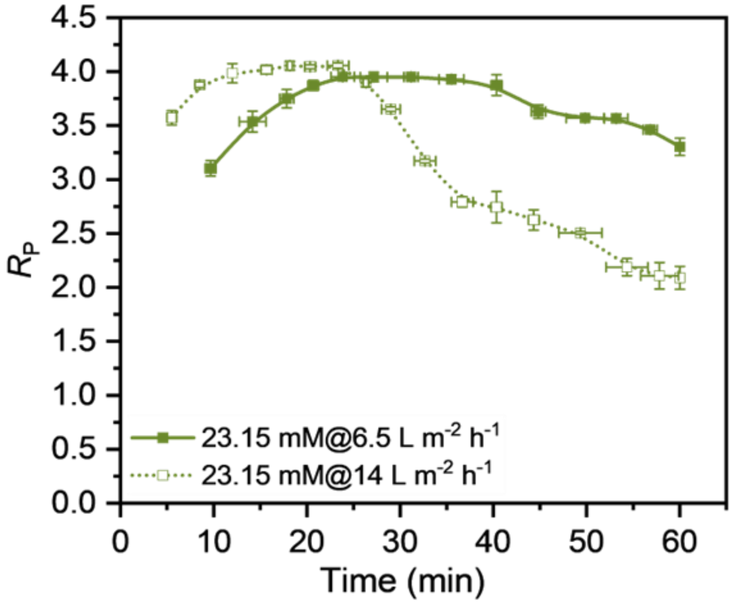

Supplementary Figure 20. The change of R_P with time using different fluxes in continuous-flow reaction for YmPh-LCI@M. The corresponding volumetric flow rates are 2.4 and 5.2 ml h^{-1}, respectively. The averaged membrane area is approx. 3.72 cm^2. Error bars represent s.d. of the mean from three independent experiments (n = 3). Source data are provided as a Source Data file.

2.4.5. Enhanced performance by tailoring BCP membrane nanochannels

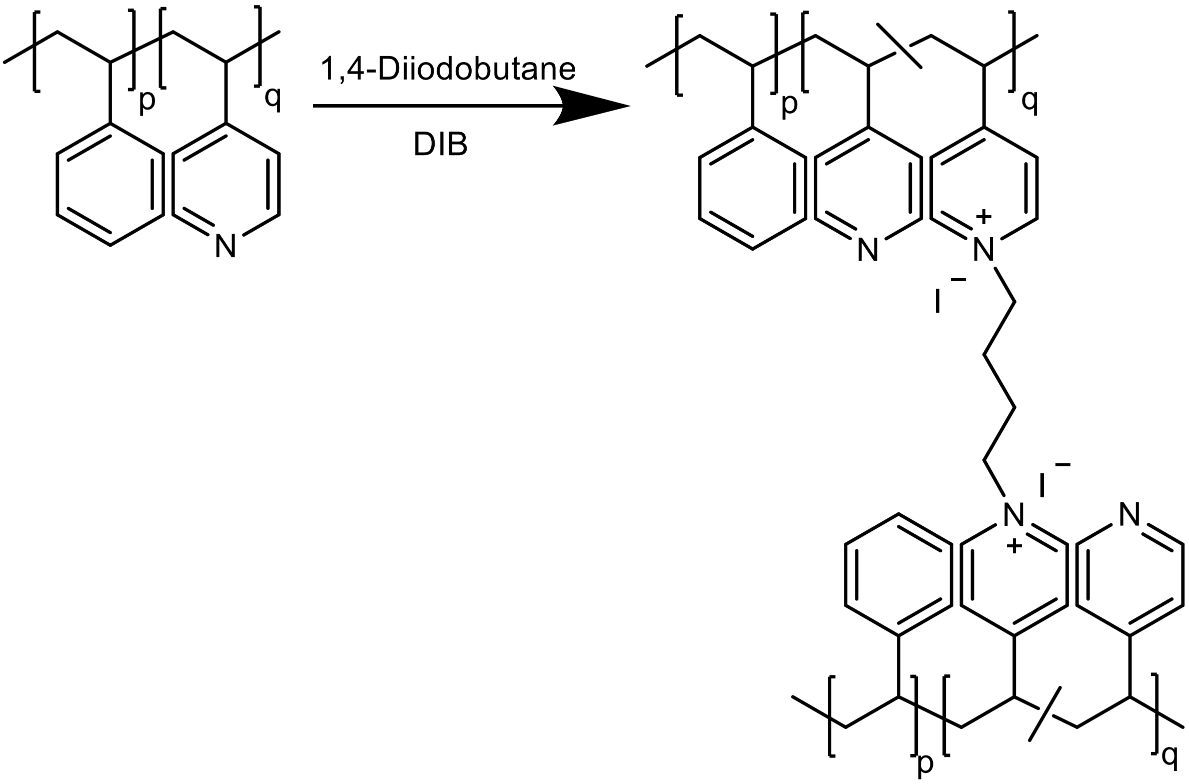

Supplementary Figure 21. Reaction scheme to introduce positive charges into P4VP pore-forming block of PS-*b*-P4VP membranes using 1,4-Diiodobutane (DIB).

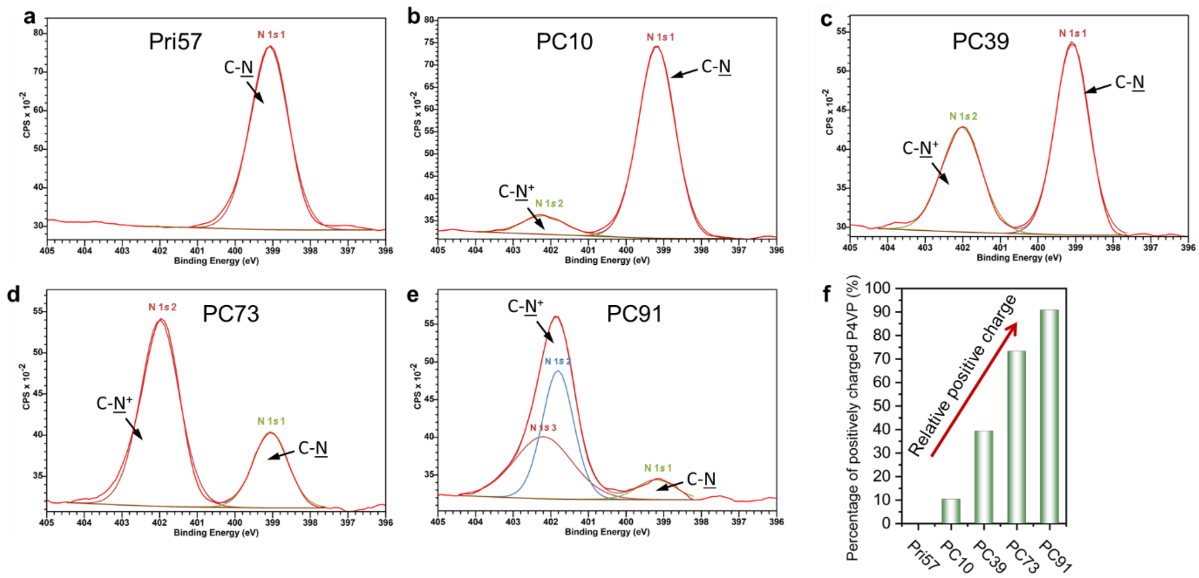

Supplementary Figure 22. Determination of percentage of positively charged P4VP in the post-modified BCP membranes. (a-e) XPS N 1*s* core-level spectra of (a) Pri57, (b) PC10, (c) PC39,

(d) PC73, and (e) PC91. (f) Content of positively charged P4VP determined by XPS. Source data are provided as a Source Data file.

The series of membranes post-modified with DIB were investigated via XPS (Supplementary Figure 22-Supplementary Figure 23). We observed a distinct nitrogen (N) signal of the pyridine rings at 399 eV binding energy in the pristine membrane (Pri57) (Supplementary Figure 22a)[18]. After quaternization, a new peak appears at 402 eV binding energy, attributed to positively charged N^+ of the pyridine rings (Supplementary Figure 22b-e)[19-22]. The area ratio of two peaks at 399 eV and 402 eV was used to estimate the percentage of positively charged P4VP in membrane nanochannels. As a result, we obtained a series of membranes with 10, 39, 73, and 91% positively charged P4VP, denoted as PC10, PC39, PC73, and PC91.

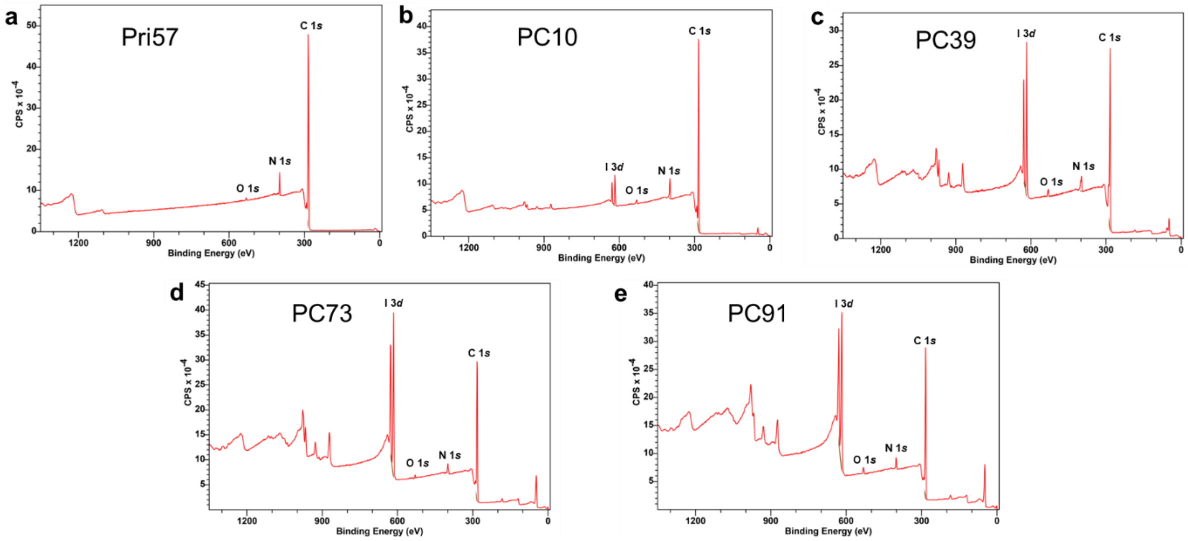

Supplementary Figure 23. XPS survey scans of (a) Pri57, (b) PC10, (c) PC39, (d) PC73, and (e) PC91. Source data are provided as a Source Data file.

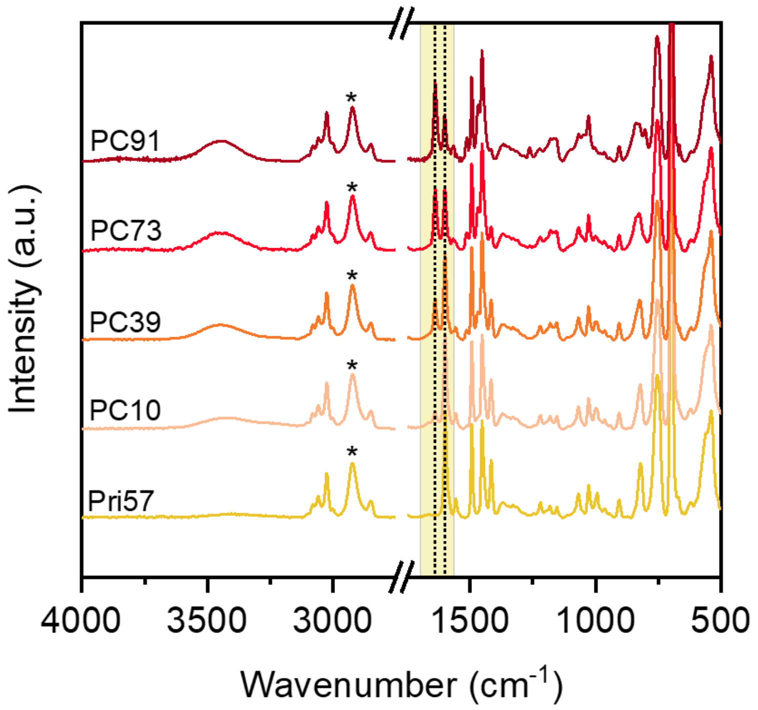

Supplementary Figure 24. ATR-FTIR spectra of Pri57, PC10, PC39, PC73, and PC91. The relative intensities were normalized to the characteristic CH_2 stretching vibration (*) of the unreactive PS-*b*-P4VP backbone around 2924 cm^{-1}. FTIR: 3400, 3026, 2924, 1639, 1601, 1493, 1452, 1167, 1029, 840, 756, 697, 540 cm^{-1}. Source data are provided as a Source Data file.

In the FTIR spectra (Supplementary Figure 24), the positively charged membranes exhibit a new characteristic vibration at ca. 1640 cm^{-1}, assigned to the stretching vibration of $C{=}N^{+}$ in positively charged 4-vinyl pyridine (4VP) moieties. The intensity of the $C{=}N^{+}$ stretching vibration increases while that of the characteristic stretching vibration of aromatic C=N and C=C at ca. 1600 cm^{-1} decreases from PC10 to PC91. The broad vibration at ca. 3400 cm^{-1} is attributed to water molecules associated with the positively charged 4VP moieties[14,23]. The FTIR results again confirm the successful introduction of positive charges in the membrane nanochannels.

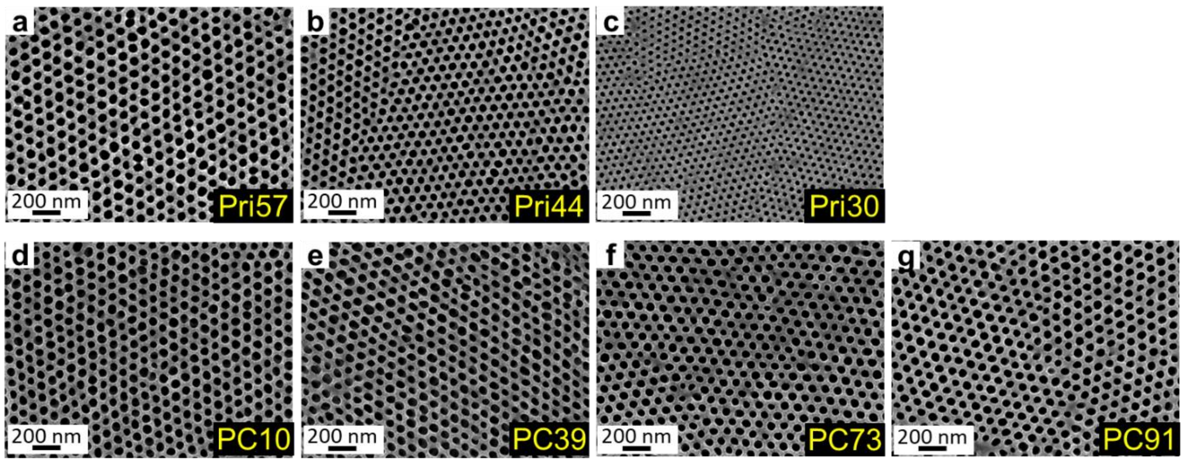

Supplementary Figure 25. SEM images of the top surface of membranes. (a) 57 nm pore size (Pri57), (b) 44 nm pore size (Pri44), (c) 30 nm pore size (Pri30), (d) PC10, (e) PC39, (f) PC73, and (g) PC91. All membranes possess the desired isoporous structure. Results were reproduced three times independently; representative micrographs are shown.

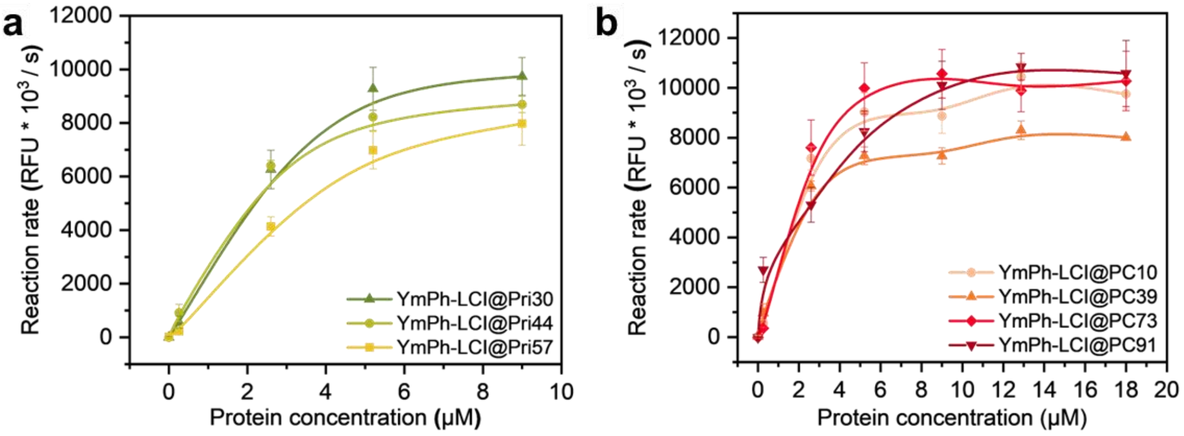

Supplementary Figure 26. Dose-response curve to analyze binding efficiency of YmPh-LCI on various tailored isoporous BCP membranes. Effect of YmPh-LCI concentration on the reaction rate after YmPh-LCI immobilization on different isoporous BCP membranes: (a) Pri57, Pri44, Pri30, (b) PC10, PC39, PC73, and PC91, determined based on the 4-MUP assay. All error bars represent s.d. of the mean from three independent experiments (n = 3). Source data are provided as a Source Data file.

We optimized the enzyme concentration to maximize the reaction rate after YmPh-LCI immobilization on various membranes Pri57, Pri44, Pri30, PC10, PC39, PC73, and PC91. As

expected, all membranes behave similarly to Pri57, with a sharp increase of the reaction rate with the enzyme concentration up to a certain value (approx. 5.2 μM), before levelling off. Therefore, we employed an enzyme concentration of 5.2 μM to prepare the YmPh-LCI functionalized membranes Pri57, Pri44, Pri30, PC10, PC39, PC73, and PC91.

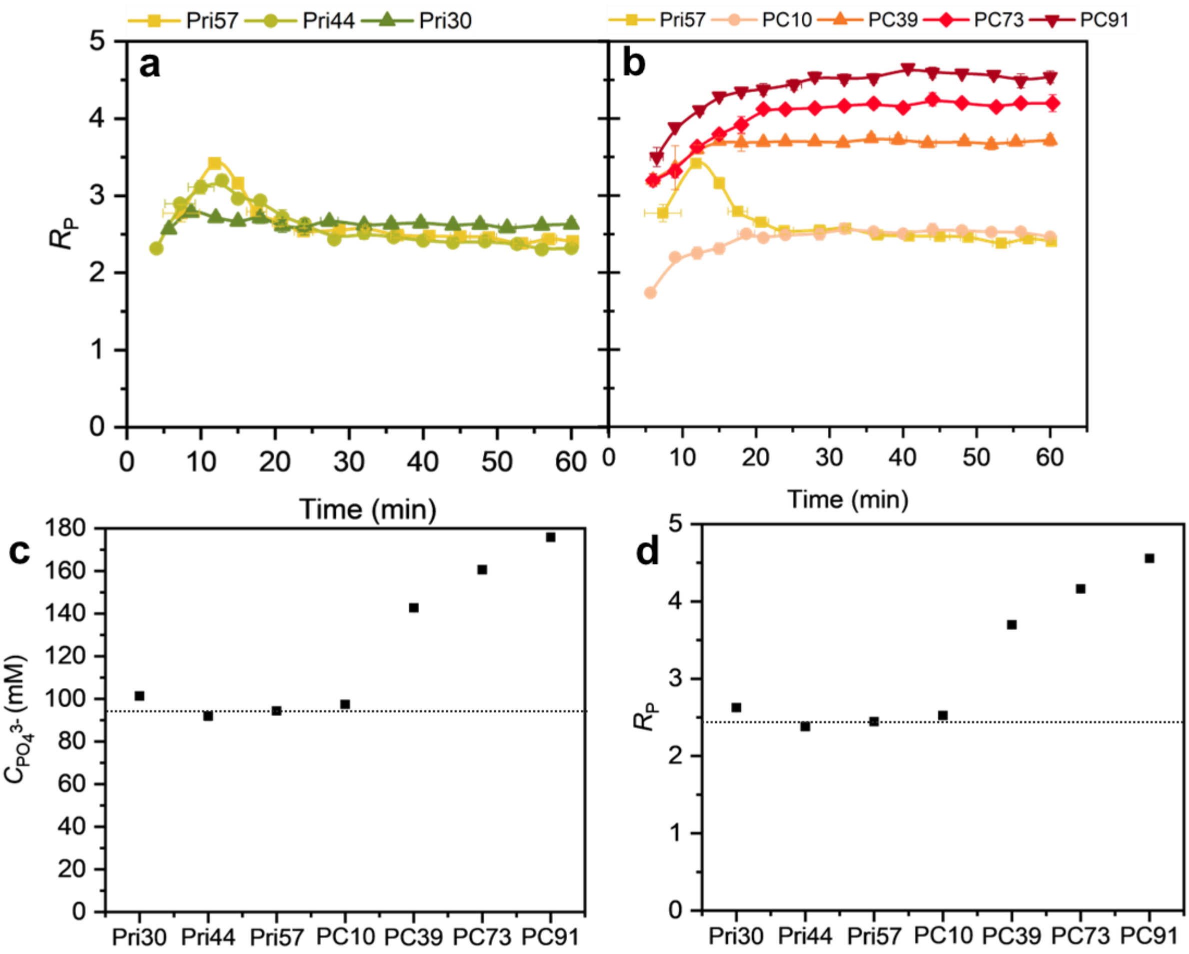

Supplementary Figure 27. Catalytic performance of tailored isoporous BCP membranes. The change of R_P with time for various YmPh-LCI immobilized membranes in a continuous-flow reaction with C_{InsP6} = 38.59 mM and flux of 6.6-7.9 L m^{-2} h^{-1} — (a) Pri57, Pri44, and Pri30; (b) Pri57, PC10, PC39, PC73, and PC91. The comparison of (c) $C_{PO_4^{3-}}$ and (d) R_P at steady state for various YmPh-LCI immobilized membranes Pri30, Pri44, Pri57, PC10, PC39, PC73, and PC91. All error bars represent s.d. of the mean from three independent experiments (n = 3). Source data are provided as a Source Data file.

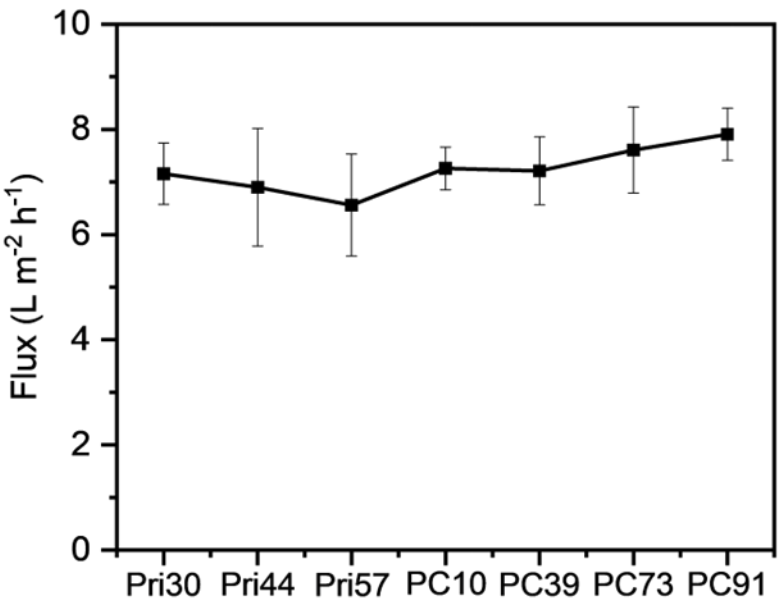

Supplementary Figure 28. Comparable flux employed in continuous-flow reaction for various YmPh-LCI immobilized membranes Pri30, Pri44, Pri57, PC10, PC39, PC73, and PC91. Error bars represent s.d. of the mean from three independent experiments (n = 3). Source data are provided as a Source Data file.

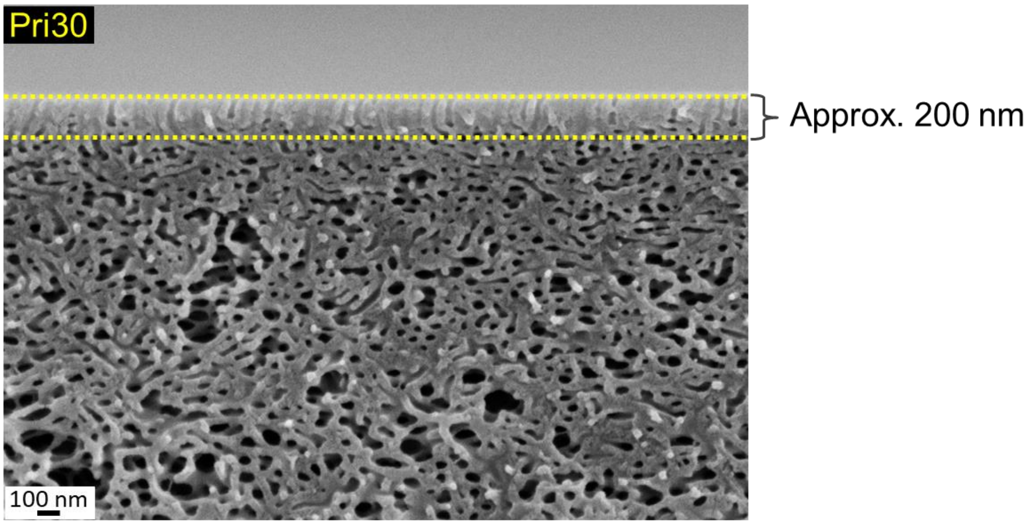

Supplementary Figure 29. SEM image of the cross-section of membrane Pri30. Results were reproduced three times independently; representative micrograph is shown.

Supplementary Figure 29 illustrates that the membrane Pri30 has an isoporous, cylindrical layer of approx. 200 nm, which is shorter than that of Pri57 (approx. 350 nm). Based on the calculation method described in Section 2.4.1 and Supplementary Table 5, the residence time inside the nanochannels of the cylindrical top layer of Pri30 is shortened to approx. 58% of that of Pri57; however, the radial molecular diffusion time inside the nanochannels of Pri30 is also shortened to approx. 28% of that of Pri57. The reduced radial molecular diffusion time is beneficial to the catalytic performance while the reduced residence time is detrimental to the catalytic performance. Overall, the experimental results prove that in the case of Pri30 a slightly improved productivity compared to Pri57 can be achieved as trade-off between these two factors influencing the catalytic performance.

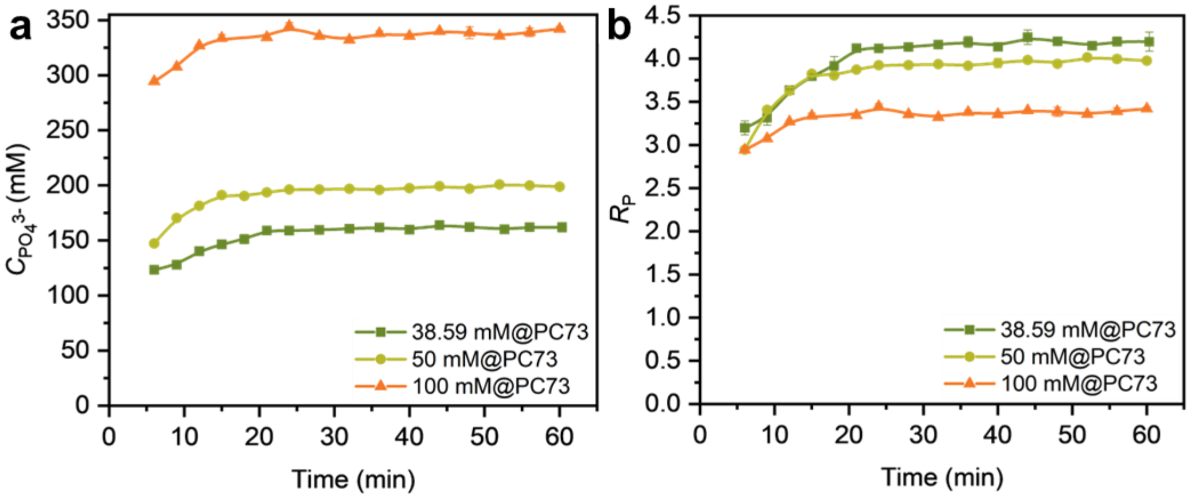

Supplementary Figure 30. Effect of C_{InsP6} on the variation of (a) $C_{PO_4^{3-}}$ and (b) R_P with time for PC73. Error bars represent s.d. of the mean from three independent experiments (n = 3). Source data are provided as a Source Data file.

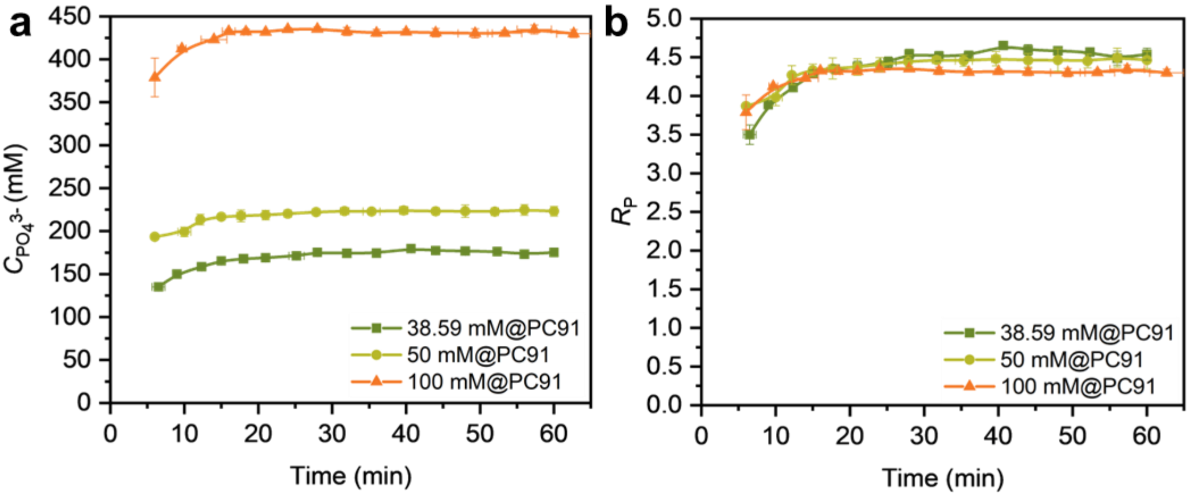

Supplementary Figure 31. Effect of C_{InsP6} on the variation of (a) $C_{PO_4^{3-}}$ and (b) R_P with time for PC91. Error bars represent s.d. of the mean from three independent experiments (n = 3). Source data are provided as a Source Data file.

2.4.6. Long-term catalytic performance of NaMeR PC91

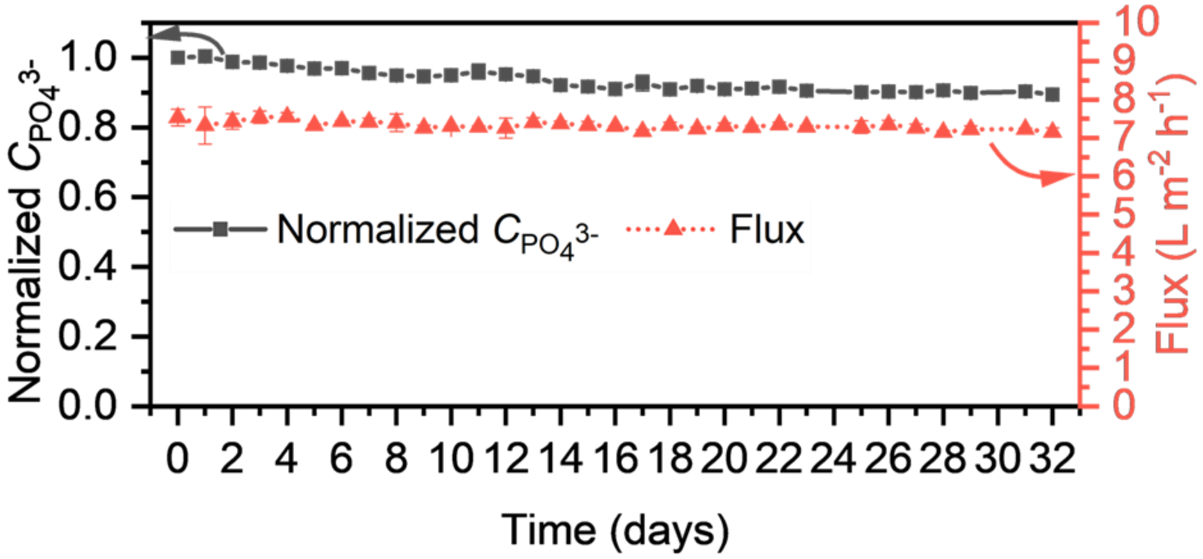

Supplementary Figure 32. Long-term stability of the continuous-flow reaction for PC91. Normalized $C_{PO_4^{3-}}$ (black curve) and flux (red curve) within 32 days of continuous-flow reaction. Error bars represent s.d. of the mean from three independent experiments (n = 3). Source data are provided as a Source Data file.

2.5. Binding efficiency of other enzymes within BCP membrane of Pri57

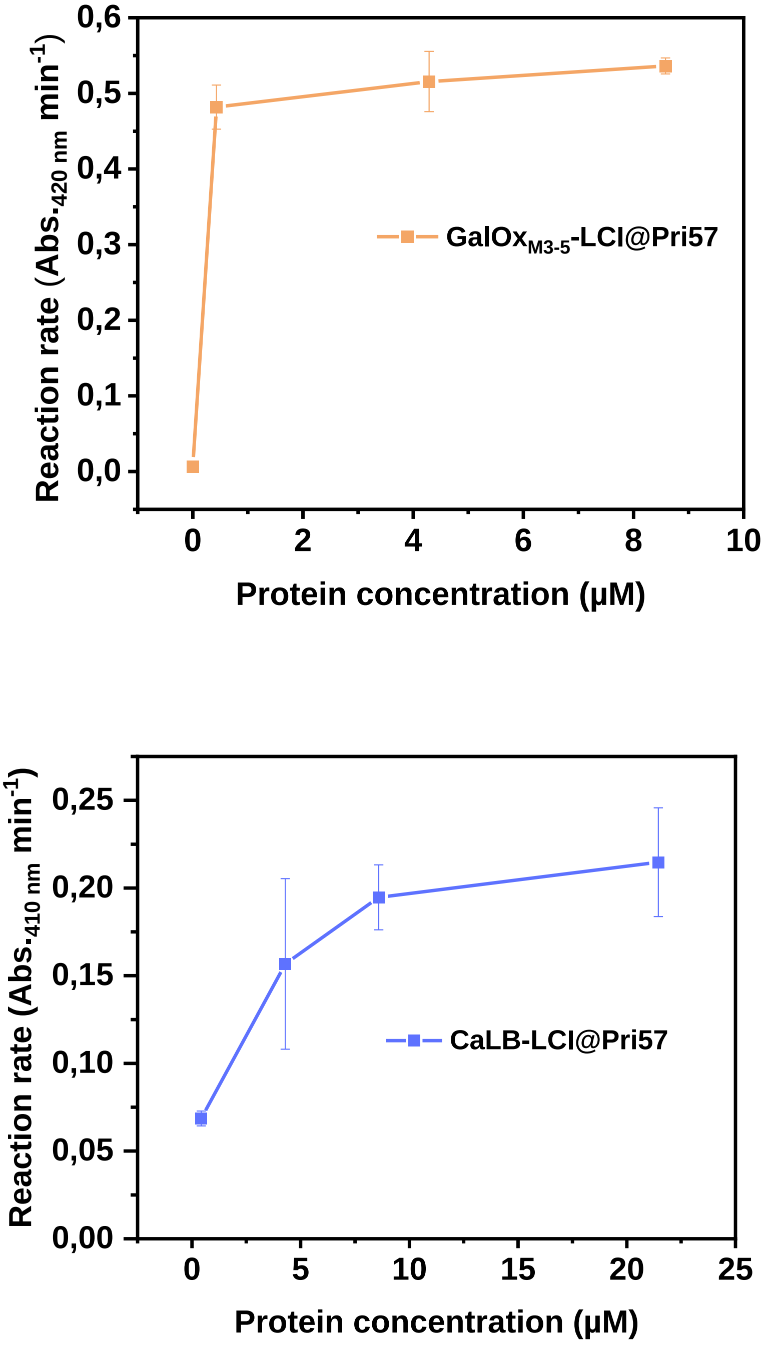

Supplementary Figure 33. Dose-response curve to analyze binding efficiency of $GalOx_{M3-5}$-LCI and CaLB-LCI on isoporous BCP membranes (Pri57). Error bars represent s.d. of the mean from three independent experiments (n = 3). Source data are provided as a Source Data file.

2.6. Synthesis of PS-*b*-P4VP diblock copolymers

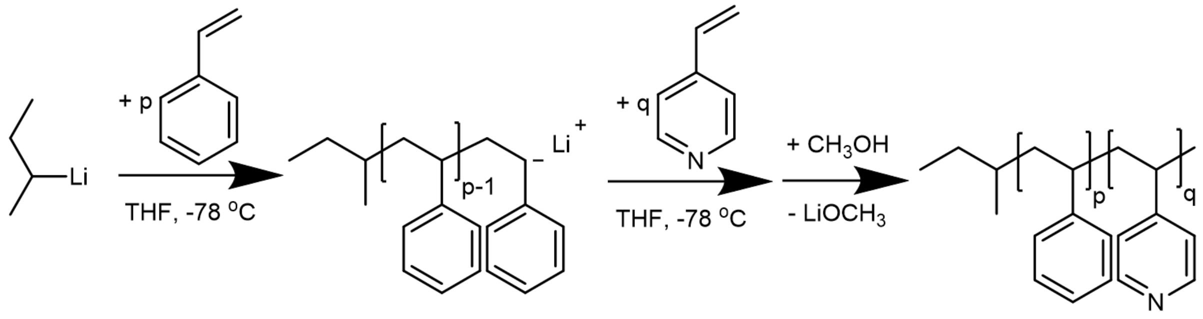

Supplementary Figure 34. Synthesis route of PS-*b*-P4VP diblock copolymers by living anionic polymerization.

3. Supplementary Tables

Supplementary Table 1. Molecular characterization of the diblock copolymers used for membrane preparation.

	PS [weight%][a)]	s	M_n [kg mol^{-1}][b)]	M_w [kg mol^{-1}][c)]	Dispersity[c)]
PS-*b*-P4VP for 57.5 nm series	76	24	238	246	1.02
PS-*b*-P4VP for 44 nm series	74	26	160	166	1.04
PS-*b*-P4VP for 30 nm series	82	18	143	154	1.08

[a)] Composition of polymers calculated from ^{1}H NMR spectra; [b)] Number averaged molecular weight calculated based on ^{1}H NMR spectra and GPC; [c)] Weight averaged molecular weight and dispersity determined by GPC.

Supplementary Table 2. Binding capacity and binding efficiency of YmPh-WT and YmPh-LCI onto isoporous BCP membranes and MTP plates made of polypropylene.

		YmPh-WT		YmPh-LCI	
Entry		BCP membrane	MTP	BCP membrane	MTP
1	C_0 (g L^{-1})	0.271 ± 0.005	0.271 ± 0.005	0.298 ± 0.004	0.298 ± 0.004
2	C_1 (g L^{-1})	0.228 ± 0.006	0.267 ± 0.009	0.155 ± 0.010	0.290 ± 0.006
3	C_{w1} (g L^{-1})	0.043 ± 0.010	0.008 ± 0.007	0.007 ± 0.005	0.006 ± 0.005
4	C_{w2} (g L^{-1})	0.017 ± 0.007	0.004 ± 0.003	0.001 ± 0.001	0.003 ± 0.002
5	C_{w3} (g L^{-1})	0.005 ± 0.007	0.002 ± 0.001	0.007 ± 0.004	0.002 ± 0.001
Binding capacity (pmol cm^{-2})		< 10	< 10	830	< 10
Binding efficiency		< 0.5%	< 0.5%	42%	< 0.5%

YmPh-WT and YmPh-LCI binding onto MTP plates (polypropylene) were negative controls to reflect the background binding of proteins on the surface of MTP wells[13], while isoporous BCP membranes were placed inside MTP wells for binding detections. The amount of YmPh-LCI binding to MTP (polypropylene) or isoporous BCP membrane (diameter 6 mm) using shaking incubation process was quantified at 280 nm using a spectrophotometer (Nanodrop™ 2000; ThermoFisher Scientific) and purified proteins (YmPh-WT: 47.3 kDa, extinction coefficient 49890 M^{-1} cm^{-1}; YmPh-LCI: 55.1 kDa, extinction coefficient 73840 M^{-1} cm^{-1}). The values were averaged from three replicates (n=3) with the standard deviation (s.d.).

After the determination of optimal YmPh-LCI immobilization concentration (C_0), the amount of membrane-immobilized enzyme was quantified. The enzyme concentration of the supernatant after immobilization (C_1) and washing solutions (C_{w1}, C_{w2}, C_{w3}) was measured by UV-vis spectrophotometry at a wavelength of 280 nm (Supplementary Table 2). Based on the protein concentration from each washing step, we calculated the amount of immobilized enzyme. The binding capacity of YmPh-LCI on the membrane was approx. 830 pmol cm^{-2} with a binding efficiency of 42%, while YmPh-WT was below the detection limit.

Supplementary Table 3. Roughness and thickness of the pristine dense film and the film after YmPh-LCI immobilization.

Roughness	Pristine dense film	after YmPh-LCI immobilization
*R*a (nm)	0.48	0.86
*R*q (nm)	0.6	1.08
Film thickness (nm)	56.5 ± 2.1	63.6 ± 0.9

*R*a: Arithmetic average of profile height deviations from the mean image plane. *R*q: Root mean square average of profile height deviations from the mean image plane. The roughness was determined based on AFM height maps, excluding the bright and swollen P4VP domains. The film thickness was measured at 6 positions of the samples.

Supplementary Table 4. Diffusion coefficient *D* of different InsP6 concentrations in NaOAc buffer (250 mM, pH 5.5) determined by PFG-NMR.

Evaluated peaks from ^{1}H NMR spectrum	Chemical shift [ppm]	0.38 mM InsP6[a)]		38.59 mM InsP6		100 mM InsP6	
		Averaged *D* [$m^2 s^{-1}$]	Standard deviation	Averaged *D* [$m^2 s^{-1}$][b)]	Standard deviation[b)]	Averaged *D* [$m^2 s^{-1}$][b)]	Standard deviation[b)]
2	4.86	No detectable signal in spectra		3.50E-10	7.70E-12	3.13E-10	4.93E-12
4	4.42			3.29E-10	2.89E-12	2.90E-10	3.05E-12
6	4.4			3.40E-10	4.47E-12	3.04E-10	4.41E-12
1, 3, 5	4.16			3.31E-10	2.06E-12	2.89E-10	1.79E-12
Overall *D* [$m^2 s^{-1}$][c)]	--	--	--	3.37E-10		2.99E-10	

[a)] The concentration of 0.38 mM was too low to detect a sufficient signal for the series of NMR spectra with increasing gradient strength *G*. [b)] The averaged *D* and standard deviation for each evaluated peak were calculated based on 10 times repeated measurements for 38.6 mM and 5 times repeated measurements for 100 mM. [c)] The overall diffusion coefficient *D* was determined by averaging the calculated *D* from all the evaluated peaks.

As expected, the diffusion coefficient of InsP6 decreases with increasing concentration, e.g., $D_{38.59mM} = 3.37 \times 10^{-10}$ m^2 s^{-1}, $D_{100mM} = 2.99 \times 10^{-10}$ m^2 s^{-1}. Although the concentration of 0.38 mM was too low to detect a sufficient signal for the series of NMR spectra with increasing gradient strength *G*, we could roughly predict that $D_{0.38mM}$ is in a similar order of magnitude (Supplementary Table 4). Therefore, we can employ $D_{38.59mM}$ and D_{100mM} to evaluate the diffusion time of substrate InsP6.

The radial molecular diffusion time (t_d) can be calculated using the equation[16]:

$$t_{\mathrm{d}} = \frac{(d_{\mathrm{p}}/2)^2}{D} \tag{4}$$

where d_p is the average pore diameter, measured with the software IMS (Imagic Bildverarbeitung AG, Opfikon, Switzerland) based on the SEM images of the top surface, and *D* is the diffusion coefficient of InsP6 as determined by PFG-NMR.

Supplementary Table 5. The membrane characteristics and the calculation of diffusion time of InsP6 in the nanochannels.

Pore diameter d_p (nm)	Porosity ε (%)	Thickness of selective layer L (nm)	Pore number density (m^{-2})	Diffusion coefficient of InsP6 D (m^2 s^{-1})	Diffusion time of InsP6 t_d (s)
57	33.5 ± 2.2	342 ± 23	1.44 ± 0.06 × 10^{14}	3.37 × 10^{-10}	2.41 × 10^{-6}
				2.99 × 10^{-10}	2.72 × 10^{-6}

Supplementary Table 6. Effect of flux on the phytase-catalyzed hydrolysis of InsP6 for YmPh-LCI@M. C_{InsP6} = 0.38 mM was used for these measurements.

Pressure (mbar)	5	10	15	20	30	40	50	60	70	100	150	10[a)]	--[b)]
Flux (L m^{-2} h^{-1})	6	15	21	32	40	53	64	75	100	151	215	15	--[b)]
Volumetric flow rate (mL h^{-1})[c)]	2.3	5.7	8.0	12.2	15.2	20.1	24.3	28.5	38.0	57.4	81.7	5.7	--[b)]
$C_{PO_4^{3-}}$ (mM)	1.88	1.89	1.81	1.77	1.73	1.64	1.6	1.5	1.47	1.32	1.26	1.72	1.83
R_P	4.9	4.9	4.7	4.6	4.5	4.3	4.1	3.9	3.8	3.4	3.3	4.5	4.8
Phosphate release efficiency (%)	82	83	79	78	76	72	70	66	64	58	55	75	80
Productivity (mmol m^{-2} h^{-1})	10.68	28.15	37.41	56.73	69.57	88.32	102.14	112.95	148.45	199.06	271.95	--	--

[a)] After the measurement with the flux of 215 L m^{-2} h^{-1}, the InsP6 hydrolysis efficiency was determined with a flux of 15 L m^{-2} h^{-1} again. [b)] Free YmPh-LCI in batch reaction with C_{InsP6} = 0.38 mM, 1875 pmol free YmPh-LCI at shaking with 90 rpm for 16 h, at room temperature (21-23 ^{o}C). [c)] The averaged membrane area used for measurements is 3.80 cm^2. Source data are provided as a Source Data file.

We calculated the volumetric flow rate based on the flux as the following equation:

$$\text{Volumetric flow rate} = J_\mathrm{p} \times A \times 10 \quad (5)$$

where the volumetric flow rate is with a unit of mL h^{-1}; J_p is the permeate flux with a unit of L m^{-2} h^{-1}; A is the top surface area of the used membrane in a unit of cm^2.

Supplementary Table 7. Effect of C_{InsP6} on the reaction efficiency of YmPh-LCI@M under optimal flux (~ 15 L m^2 h^1) and free YmPh-LCI in batch reaction (enzyme amount 1000 pmol, 18 h reaction).

C_{InsP6} (mM)	0.04	0.08	0.19	0.38	0.77	1.93	3.86	7.72	11.58	15.43	23.15	30.87	38.59
Flux (L m^{-2} h^{-1})	15	15	16	15	15	16	16	15	14.5	14	14.5	13	13
Volumetric flow rate (mL h^{-1})[a)]	5.6	5.6	5.9	5.6	5.6	5.9	5.9	5.6	5.4	5.2	5.4	4.8	4.8
$C_{PO_4^{3-}}$ (mM)	0.21	0.38	0.94	1.77	3.51	8.34	15.62	31.76	48.30	62.10	93.81	117.67	119.11
R_P	5.4	5.0	4.9	4.6	4.5	4.3	4.0	4.1	4.2	4.0	4.1	3.8	3.1
Phosphate release efficiency (%)	88	79	82	78	76	72	67	69	70	67	68	64	51
$C_{PO_4^{3-}}$ (mM)[b)]	0.22	0.40	0.95	1.80	3.53	8.66	17.71	32.74	54.13	67.69	95.25	139.17	161.54
R_P[b)]	5.6	5.2	4.9	4.7	4.6	4.5	4.6	4.2	4.7	4.4	4.1	4.5	4.2
Phosphate release efficiency (%)[b)]	92	83	83	79	76	75	76	71	78	73	69	75	70

[a)] The averaged membrane area used for measurements is 3.71 cm^2. [b)] Free YmPh-LCI batch reactions used 1000 pmol enzyme, 1 mL InsP6 solutions, and 18 h incubation at 90 rpm shaking, room temperature (21-23 ^{o}C). Source data are provided as a Source Data file.

Supplementary Table 8. Self-hydrolysis of InsP6 in NaOAc buffer (250 mM, pH 5.5).

C_{InsP6} (mM)	0.04	0.08	0.19	0.38	0.77	1.93	3.86	7.72	11.58	15.43	23.15	30.87	38.59
$C_{PO_4^{3-}}$ (mM) from self-hydrolysis	0.009	0.015	0.032	0.060	0.077	0.004	0.002	0.002	0.0				

Note: The small amount of self-hydrolysis was neglected for determining the hydrolysis efficiency in flow reactions.

Supplementary Table 9. Comparison of maximal phosphate concentration ($C_{PO_4^{3-}}$) at steady state conditions with immobilized YmPh-LCI in membranes Pri30, Pri44, Pri57, PC10, PC39, PC73, and PC91.

Membrane	Pri30	Pri44	Pri57	PC10	PC39	PC73	PC91
$C_{PO_4^{3-}}$(mmol L^{-1})	101 ± 0.9	92 ± 2.1	94 ± 1.5	97 ± 1.3	143 ± 1.2	161 ± 1.4	176 ± 1.8
Flux (L m^{-2} h^{-1})	7.2 ± 0.58	6.9 ± 1.12	6.6 ± 0.97	7.3 ± 0.40	7.2 ± 0.65	7.6 ± 0.82	7.9 ± 0.49
Volumetric flow rate (mL h^{-1}) [a)]	2.6	2.6	2.5	2.7	2.7	2.8	2.8

All the $C_{PO_4^{3-}}$ are averaged values obtained under steady state conditions in a 1 hour continuous-flow reaction with an InsP6 concentration (C_{InsP6}) of 38.59 mM; see also Supplementary Figure 27. The averaged membrane areas used for measurements is in the range of 3.59-3.80 cm^2. Source data are provided as a Source Data file.

Supplementary Table 10. Comparison of maximal phosphate concentration ($C_{PO_4^{3-}}$) at steady state for the PC73 and PC91 with varied $InsP_6$ concentration (C_{InsP6} of 38.59, 50.00, and 100.00 mM).

	PC73			PC91		
C_{InsP6} (mmol L^{-1})	38.59	50.00	100.00	38.59	50.00	100.00
$C_{PO_4^{3-}}$(mmol L^{-1})	161 ± 1.4	198 ± 1.8	338 ± 1.4	176 ± 1.8	223 ± 4.7	434 ± 1.5
Flux (L m^{-2} h^{-1})	7.6 ± 0.82	7.9 ± 0.15	7.9 ± 0.25	7.9 ± 0.49	8.0 ± 0.40	7.5 ± 0.22
Volumetric flow rate (mL h^{-1}) [a)]	2.8	2.9	2.9	2.9	2.9	2.8

All the $C_{PO_4^{3-}}$ are averaged values obtained under steady state conditions in 1 hour continuous-flow reactions; see also Figure 5d, e in the main manuscript. [a)] The averaged membrane area used for measurements is 3.68 cm^2 of PC73, and 3.65 cm^2 of PC91. Source data are provided as a Source Data file.

Supplementary Table 11. The calculation of maximal theoretical productivity of PC91 within 30 days of continuous-flow reaction.

C_{InsP6} (mmol L^{-1})	$C_{PO_4^{3-}}$ (mmol L^{-1})[a)]	R_P [a)]	Flux J_p (L m^{-2} h^{-1})[a)]	Volumetric flow rate (mL h^{-1}) [b)]	Productivity (mmol m^{-2} h^{-1}) [c)]	Theoretical Productivity (mol per m^2 membrane within 30 days) [d)]	Actual Productivity (mol per m^2 membrane within 30 days) [e)]	Actual / Theoretical Productivity (%)
100	434.3 ± 1.5	4.34	7.5 ± 0.22	2.7	3270	2354	2147	91.2

[a)] $C_{PO_4^{3-}}$ and J_p are the averaged values of steady state during 1 hour continuous-flow reaction with C_{InsP6} = 100 mM in Supplementary Figure 30. [b)] The averaged membrane area used for measurements is 3.61 cm^2. [c)] Productivity within 1 hour continuous-flow reaction is calculated based on the obtained $C_{PO_4^{3-}}$ and J_p at the steady state. [d)] The theoretical productivity within 30 days can be calculated based on the productivity within 1 hour at the steady state assuming no loss of the catalytic performance within 30 days. [e)] Actual productivity within 30 days is calculated based on the obtained $C_{PO_4^{3-}}$ and J_p in Supplementary Figure 31. Source data are provided as a Source Data file.

Under assumption of no loss of catalytic performance over 30 days continuous flow reaction, the maximal theoretical productivity within 30 days was calculated by the following equation:

$$\text{Theoretical Productivity within 30 days} = C_{\mathrm{PO_4}^{3-}} \times J_p \times 24 \times 30 \qquad (6)$$

where $C_{PO_4^{3-}}$ is the concentration of phosphate in the permeate solution with a unit of mM and J_p is the permeate flux with a unit of L m^{-2} h^{-1} at the steady state within 1 hour continuous flow reaction.

Supplementary Table 12. The calculation of space-time yield (STY) of immobilized YmPh-LCI of PC91 during 32 days of continuous-flow reaction.

$C_{PO_4^{3-}}$ (mmol L^{-1})[a)]	J_p (L m^{-2} h^{-1})[a)]	Volumetric flow rate (mL h^{-1})[b)]	V_r (L m^{-2})	$M_{PO_4^{3-}}$ (g mol^{-1})	STY (g L^{-1} d^{-1})	Enzyme amount (µmol m^{-2})
406 ± 14	7.3 ± 0.1	2.6	6.47×10^{-2}	94.97	1.05×10^{5}	15.02 ± 1.6

[a)] $C_{PO_4^{3-}}$ and J_p are the averaged values from 32 days of continuous-flow reaction in Fig. 6a, the main manuscript. [b)] The averaged membrane area used for measurements is 3.61 cm^2. Source data are provided as a Source Data file.

For reactor comparison purposes, in this work, space-time yield was also applied, adapted from the previous study. In this work, space-time yield (STY) is defined as the amount of product phosphate hydrolysed per reactor volume per time, calculated based on the following equation:

$$STY = \frac{m_{PO_4^{3-}}}{V_r \times t_r} = \frac{C_{PO_4^{3-}} \times V_r \times M_{PO_4^{3-}}}{V_r \times {V_r}/{J_p}} = \frac{C_{PO_4^{3-}} \times J_p \times M_{PO_4^{3-}}}{V_r} \quad (7)$$

Where $m_{PO_4^{3-}}$ is the mass of phosphate in the permeate solution, $C_{PO_4^{3-}}$ is the concentration of phosphate in the permeate solution, $M_{PO_4^{3-}}$ is the molecular weight of phosphate groups, J_p is the permeate flux, t_r is the time for the molecules passing through the whole membrane layer, and V_r is the pore volume of the whole membrane layer per unit of membrane surface area, which is determined by mercury-intrusion porosimetry using AutoPore V mercury intrusion porosimeter (Micromeritics Instrument Corporation, Norcross, Georgia, USA)[24].

Supplementary Table 13. Quantification of reactivity of free YmPh-LCI and YmPh-LCI@M in Pri57 membrane.

Entry		Reactivity (RFU s^{-1} $pmol_{enzyme}^{-1}$)
1	Free YmPh-LCI	990
2	YmPh-LCI@M	0.031

The reactivity of free YmPh-LCI was calculated from the reaction rate of 8.49×10^4 RFU s^{-1} with 0.858 µM enzyme (100 µl, Supplementary Figure 3); The reactivity of YmPh-LCI@M of Pri57 was calculated from the reaction rate of 6.98 RFU s^{-1} with 830 pmol cm^{-2} immobilized enzyme (0.28 cm^2 membrane, Supplementary Figure 5b, Supplementary Table 2).

Supplementary Table 14. Quantification of the sodium and water in the InsP6 substrate.

Components	P	Na	ddH_2O
Compositions (mg/g_{InsP6})	217.3	153.8	74.5

10 mg InsP6 sample was dissolved in 10 mL ddH_2O freshly before detection. After further dilution, phosphorus and sodium were determined by atomic emission spectroscopy (High-Resolution Array ICP-OES) (PlasmaQuant® PQ9000 Elite, Analytik Jena AG, Germany; software Aspect PQ version 1.2.4.0). Afterwards, water content was calculated based on the sample molecular structure $C_6H_{18}O_{24}P_6 \cdot xNa \cdot yH_2O$. The determined molecular structure is $C_6H_{18}O_{24}P_6 \cdot 5.72Na \cdot 3.53H_2O$ with a molecular weight of 855.25 g mol^{-1}.

4. Supplementary Notes

4.1 Plasmid map of YmPh-17Helix-TEV-LCI:

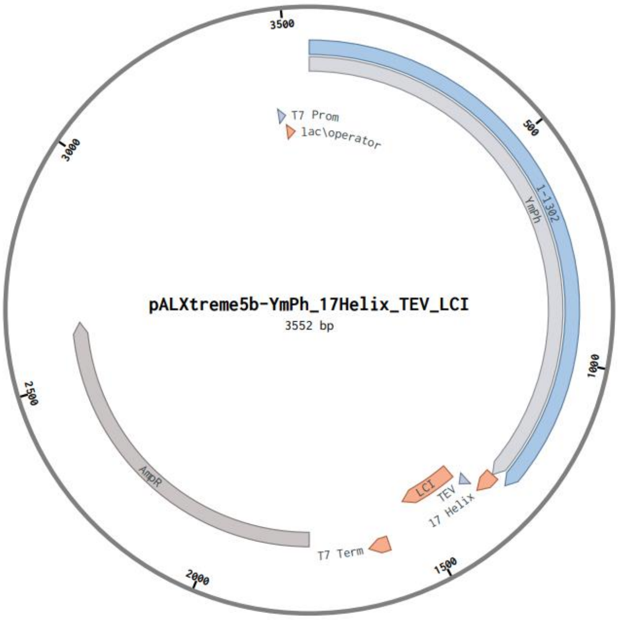

4.2 DNA sequence of YmPh-17Helix-TEV-LCI:

ATGCGATTAACTGCACTGGGCTTAATGCTAGGTAGTTTTGCGATCAGTGCTGCAC

CAGTTGCTGCACCAGTGACGGGTTACACTTTGGAGCGAGTGGTTATCTTGAGCCG

CCATGGTGTTCGTTCGCCGACGAAACAAACAGAGTTAATGAATGATGTCACACC

GGATAAGTGGCCTCAATGGCCGGTACCAGCGGGGTATTTAACCCCCAGAGGTGC

GCAATTAGTGACACTGATGGGCGGCTTCTATGGTGATTATTTCCGCAATCAAGGA

TTGCTCCCGGCAGGATGCCCGGCAGATGGAACCCTCTATGCGCAAGCCGATATC

GATCAACGAACCCGTTTAACTGGGCAAGCATTCCTTGATGGCATAGCTCCGGGGT

GCGGTCTAAAGGTGCATTATCAGGCTGATTTGAAAAAGGTTGATCCACTGTTTCA

CCCCGTCGAAGCCGGTGTGTGTCAGCTAGATTCGACACAAACCCATAGGGCCATT

GAGGCGCAACTGGGGGCGCCATTAAGTGAACTTAGCCAGCGTTATGCTAAGCCA

TTTGCCCAGATGGGCGAGATTCTCAATTTCACTGCTTCCCCCTATTGCAAGTCACT

ACAGCAACAAGGAAAATCCTGTGATTTTGCCACCTTTGCTGCCAATGAAGTTAAG

GTGAATCAGCAGGGGACAAAGGTATCGCTCAGTGGGCCGCTGGCACTCTCATCC

ACATTGGGTGAAATCTTCTTGCTACAAAATTCGCAAGGGATGCCGGATGTCGCTT

GGCATCGATTAAGTGGAGCGGAAAATTGGGTCTCATTATTATCGCTGCATAATGC

GCAATTTGATTTGATGGCTAAAACACCTTATATCGCCCGTCATAAGGGAACTCCG

TTGTTGCAACAGATTGTGACGGCGCTAGTGCTTCAGCGTAAGGGGCAAGGCCAA

ACTTTGCCATTATCTGAGCAGACCAAACTCCTTTTCCTTGGCGGTCATGATACCA

ATATCGCCAATATTGGCGGTATGCTAGGAGCCAACTGGCAGCTACCGCAACAGC

CCGATAACACCCCGCCGGGTGGGGGCTGGTGTTTGAACTATGGCAGAATCCAG

ATAACCATCAGCAATATGTCGCAGTTAAGATGTTCTATCAAACAATGGATCAGTT

ACGAAATAGTGAAAAGTTAGACCTGAAAAGTCATCCAGCCGGTATTGTTCCCATT

GAGATCGAAGGTTGTGAGAACATCGGTACAGACAAACTTTGCCAGCTTGATACC

TTCCAAAAGAGAGTGGCTCAGGTGATTGAACCTGCATGCCATATTGCAGAAGCA

GCAGCAAAAGAAGCCGCTGCCAAAGAAGCGGCAGCGAAAGCAGAAAATCTGTA

TTTTCAGGGTGCCATTAAACTGGTTCAGAGCCCGAATGGTAATTTTGCAGCAAGC

TTTGTTCTGGATGGCACCAAATGGATCTTCAAAAGCAAATACTATGACAGCAGCA

AAGGTTATTGGGTGGGTATTTATGAAGTGTGGGATCGCAAA

4.3 Amino acid sequence of YmPh-17Helix-TEV-LCI:

MRLTALGLMLGSFAISAAPVAAPVTGYTLERVVILSRHGVRSPTKQTELMNDVTPDK

WPQWPVPAGYLTPRGAQLVTLMGGFYGDYFRNQGLLPAGCPADGTLYAQADIDQR

TRLTGQAFLDGIAPGCGLKVHYQADLKKVDPLFHPVEAGVCQLDSTQTHRAIEAQL

GAPLSELSQRYAKPFAQMGEILNFTASPYCKSLQQQGKSCDFATFAANEVKVNQQG

TKVSLSGPLALSSTLGEIFLLQNSQGMPDVAWHRLSGAENWVSLLSLHNAQFDLMA

KTPYIARHKGTPLLQQIVTALVLQRKGQGQTLPLSEQTKLLFLGGHDTNIANIGGML

GANWQLPQQPDNTPPGGGLVFELWQNPDNHQQYVAVKMFYQTMDQLRNSEKLDL

KSHPAGIVPIEIEGCENIGTDKLCQLDTFQKRVAQVIEPACHIAEAAAKEAAAKEAAA

KAENLYFQGAIKLVQSPNGNFAASFVLDGTKWIFKSKYYDSSKGYWVGIYEVWDRK

4.4 Plasmid map of $GalOx_{M3\text{-}5}$-17Helix-TEV-LCI:

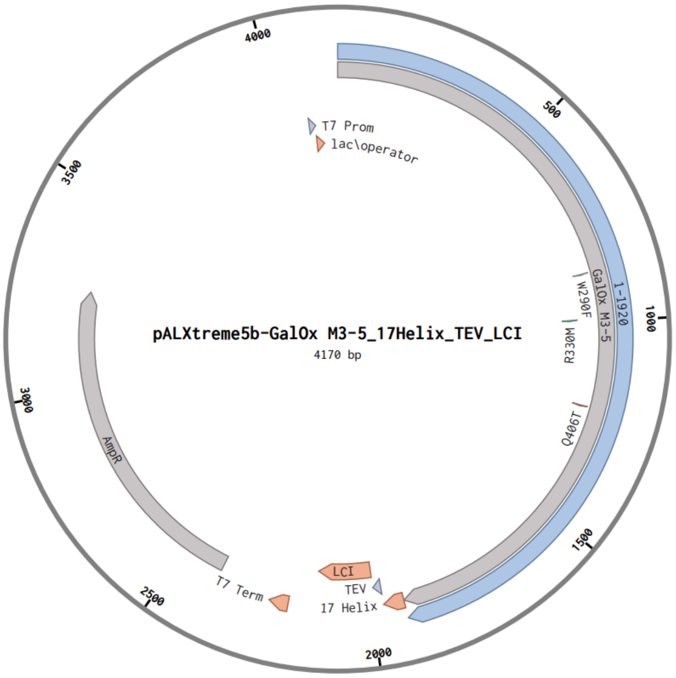

4.5 DNA sequence of $GalOx_{M3\text{-}5}$-17Helix-TEV-LCI:

ATGGCAAGCGCACCGATTGGTAGCGCAATTCCGCGTAATAATTGGGCAGTTACCT

GTGATAGCGCACAGAGCGGTAATGAATGTAATAAAGCCATTGATGGCAACAAGG

ATACCTTTTGGCATACCTTTTATGGTGCAAATGGTGATCCGAAACCGCCTCATAC

CTATACCATTGATATGAAAACCACGCAGAATGTGAATGGTCTGAGCGTTCTGCCT

CGTCAGGATGGTAATCAGAATGGTTGGATTGGTCGTCATGAAGTTTATCTGAGCA

GTGATGGCACCAATTGGGGTAGTCCGGTTGCAAGCGGTAGCTGGTTTGCAGATA

GCACCACCAAATATAGCAATTTTGAAACCCGTCCGGCACGTTATGTTCGTCTGGT

TGCAATTACCGAAGCAAATGGTCAGCCGTGGACCAGCATTGCAGAAATTAATGT

TTTTCAGGCAAGCAGCTATACCGCACCGCAGCCTGGTTTAGGTCGTTGGGGTCCG

ACCATTGATCTGCCGATTGTTCCGGCAGCAGCAGCAATTGAACCGACCAGCGGTC

GTGTTCTGATGTGGTCAAGCTATCGTAATGATGCATTTGAAGGTAGCCCTGGTGG

TATTACCCTGACCAGCAGCTGGGATCCGAGCACCGGTATTGTTAGCGATCGTACC

GTTACCGTGACCAAACACGACATGTTTTGTCCGGGTATTAGCATGGATGGCAATG

GTCAGATTGTTGTTACCGGTGGCAATGATGCAAAAAAAACCAGCCTGTATGATA

GCAGCAGCGATAGCTGGATTCCGGGTCCTGATATGCAGGTTGCACGTGGTTATCA

GAGCAGCGCAACCATGAGTGATGGTCGTGTTTTTACCATTGGTGGTAGTTTCAGC

GGTGGTGTTTTTGAGAAAAATGGTGAAGTGTATAGCCCGAGCAGCAAAACCTGG

ACCAGCCTGCCGAATGCAAAAGTTAATCCGATGCTGACCGCAGATAAACAGGGT

CTGTATATGAGCGATAATCATGCATGGCTGTTTGGTTGGAAAAAAGGTAGCGTGT

TTCAGGCAGGTCCGAGTACCGCAATGAATTGGTATTACACCAGCGGTAGCGGTG

ATGTTAAAAGCGCAGGTAAACGTCAGAGCAATCGTGGTGTTGCACCGGATGCAA

TGTGTGGTAATGCAGTTATGTATGATGCCGTGAAAGGTAAAATTCTGACCTTTGG

TGGTTCACCGGATTATACGGATAGTGATGCAACCACCGATGCACATATTATCACC

CTGGGTGAACCGGGTACAAGCCCGAATACCGTTTTTGCAAGCAATGGTCTGTATT

TTGCACGTACCTTTCATACCAGCGTTGTTCTGCCGGATGGTAGCACCTTTATCACC

GGTGGTCAGCGTCGTGGTATTCCGTTTGAAGATAGTACACCGGTTTTTACACCGG

AAATTTATGTTCCAGAACAGGACACCTTCTATAAACAGAATCCGAATAGCATTGT

GCGTGCCTATCATAGCATTAGCCTGCTGTTACCGGATGGTCGCGTGTTTAATGGT

GGTGGTGGCCTGTGTGGTGATTGTACCACAAATCATTTTGATGCCCAGATTTTCA

CCCCGAACTATCTGTATGATTCAAATGGTAATCTGGCGACCCGTCCGAAAATTAC

CCGTACCAGCACACAGAGCGTTAAAGTTGGTGGTCGTATTACCATTAGCACCGAT

AGCAGTATTAGCAAAGCAAGCCTGATTCGTTATGGTACAGCAACCCATACCGTTA

ATACCGATCAGCGTCGCATTCCGCTGACACTGACCAATAATGGTGGCAATAGCTA

TAGTTTTCAGGTTCCGAGCGATAGCGGTGTTGCCCTGCCTGGTTATTGGATGCTGT

TTGTTATGAATAGTGCCGGTGTGCCGAGCGTTGCAAGCACCATTCGTGTTACCCA

AGCAGAAGCAGCAGCAAAAGAAGCCGCTGCCAAAGAAGCGGCAGCGAAAGCAG

AAAATCTGTATTTTCAGGGTGCCATTAAACTGGTTCAGAGCCCGAATGGTAATTT

TGCAGCAAGCTTTGTTCTGGATGGCACCAAATGGATCTTCAAAAGCAAATACTAT

GACAGCAGCAAAGGTTATTGGGTGGGTATTTATGAAGTGTGGGATCGCAAA

4.6 Amino acid sequence of $GalOx_{M3\text{-}5}$-17Helix-TEV-LCI:

MASAPIGSAIPRNNWAVTCDSAQSGNECNKAIDGNKDTFWHTFYGANGDPKPPHTY

TIDMKTTQNVNGLSVLPRQDGNQNGWIGRHEVYLSSDGTNWGSPVASGSWFADST

TKYSNFETRPARYVRLVAITEANGQPWTSIAEINVFQASSYTAPQPGLGRWGPTIDLPI

VPAAAAIEPTSGRVLMWSSYRNDAFEGSPGGITLTSSWDPSTGIVSDRTVTVTKHDM

FCPGISMDGNGQIVVTGGNDAKKTSLYDSSSDSWIPGPDMQVARGYQSSATMSDGR

VFTIGGSFSGGVFEKNGEVYSPSSKTWTSLPNAKVNPMLTADKQGLYMSDNHAWLF

GWKKGSVFQAGPSTAMNWYYTSGSGDVKSAGKRQSNRGVAPDAMCGNAVMYDA

VKGKILTFGGSPDYTDSDATTDAHIITLGEPGTSPNTVFASNGLYFARTFHTSVVLPD

GSTFITGGQRRGIPFEDSTPVFTPEIYVPEQDTFYKQNPNSIVRAYHSISLLLPDGRVFN

GGGGLCGDCTTNHFDAQIFTPNYLYDSNGNLATRPKITRTSTQSVKVGGRITISTDSSI

SKASLIRYGTATHTVNTDQRRIPLTLTNNGGNSYSFQVPSDSGVALPGYWMLFVMNS

AGVPSVASTIRVTQAEAAAKEAAAKEAAAKAENLYFQGAIKLVQSPNGNFAASFVL

DGTKWIFKSKYYDSSKGYWVGIYEVWDRK

4.7 Plasmid map of CaLB-17Helix-TEV-LCI:

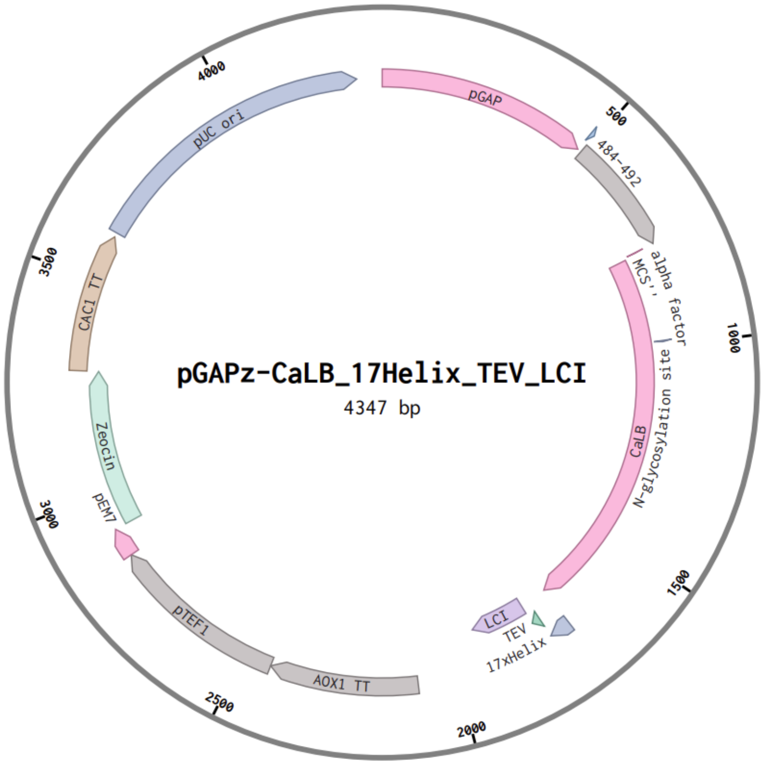

4.8 DNA sequence of CaLB-17Helix-TEV-LCI:

TTGCCATCTGGTTCTGATCCAGCTTTTTCTCAACCTAAATCTGTTTTGGATGCTGG

TTTGACTTGTCAAGGTGCTTCTCCATCTTCTGTTTCTAAGCCAATTTTGTTGGTTCC

AGGTACTGGTACTACTGGTCCACAATCTTTTGATTCTAACTGGATTCCATTGTCCA

CTCAATTGGGTTATACTCCATGTTGGATTTCTCCACCACCATTCATGTTGAACGAT

ACTCAAGTTAACACCGAATATATGGTTAACGCTATTACTGCCTTGTATGCTGGTT

CTGGTAACAACAAATTGCCAGTTTTGACTTGGTCACAAGGTGGTTTGGTTGCTCA

ATGGGGTTTGACATTTTTCCCATCTATCAGATCCAAGGTCGATAGATTGATGGCT

TTTGCTCCAGATTACAAGGGTACTGTTTTGGCTGGTCCATTGGATGCTTTGGCTGT

TTCTGCTCCATCTGTTTGGCAACAAACTACTGGTTCTGCTTTGACTACTGCTTTGA

GAAATGCTGGTGGTTTGACACAAATAGTTCCAACTACAAACTTGTACTCTGCTAC

CGACGAAATTGTCCAACCACAAGTTTCTAATTCCCCATTGGATTCCTCTTACTTGT

TCAACGGTAAGAACGTTCAAGCTCAAGCTGTTTGTGGTCCTTTGTTCGTTATTGAT

CATGCAGGTTCTTTGACCTCCCAATTCTCTTATGTTGTTGGTAGATCAGCTTTGAG

ATCTACTACAGGTCAAGCTAGATCTGCTGATTATGGTATTACTGATTGCAACCCA

TTGCCAGCTAATGATTTGACTCCAGAACAAAAAGTTGCTGCTGCAGCTTTGTTAG

CTCCAGCTGCTGCTGCTATAGTTGCTGGTCCAAAGCAAAATTGTGAACCAGATTT

GATGCCATACGCTAGACCATTTGCTGTTGGTAAAAGAACTTGTTCTGGTATCGTT

ACCCCAGCTGAAGCTGCAGCCAAAGAAGCCGCTGCCAAAGAAGCGGCAGCGAA

AGCAGAAAATCTGTATTTTCAGGGTGCCATTAAACTGGTTCAGAGCCCGAATGGT

AATTTTGCAGCAAGCTTTGTTCTGGATGGCACCAAATGGATCTTCAAAAGCAAAT

ACTATGACAGCAGCAAAGGTTATTGGGTGGGTATTTATGAAGTGTGGGATCGCA

AA

4.9 Amino acid sequence of CaLB-17Helix-TEV-LCI:

LPSGSDPAFSQPKSVLDAGLTCQGASPSSVSKPILLVPGTGTTGPQSFDSNWIPLSTQL

GYTPCWISPPPFMLNDTQVNTEYMVNAITALYAGSGNNKLPVLTWSQGGLVAQWG

LTFFPSIRSKVDRLMAFAPDYKGTVLAGPLDALAVSAPSVWQQTTGSALTTALRNAG

GLTQIVPTTNLYSATDEIVQPQVSNSPLDSSYLFNGKNVQAQAVCGPLFVIDHAGSLT

SQFSYVVGRSALRSTTGQARSADYGITDCNPLPANDLTPEQKVAAAALLAPAAAAIV

AGPKQNCEPDLMPYARPFAVGKRTCSGIVTPAEAAAKEAAAKEAAAKAENLYFQG

AIKLVQSPNGNFAASFVLDGTKWIFKSKYYDSSKGYWVGIYEVWDRK

5. Supplementary References

1 Shivange, A. V. *et al.* Directed evolution of a highly active Yersinia mollaretii phytase. *Appl. Microbiol. Biotechnol.* **95**, 405-418, doi:10.1007/s00253-011-3756-7 (2012).

2 Dedisch, S. *et al.* Matter-tag: A universal immobilization platform for enzymes on polymers, metals, and silicon-based materials. *Biotechnol. Bioeng.* **117**, 49-61, doi:https://doi.org/10.1002/bit.27181 (2020).

3 Körfer, G. *et al.* Directed evolution of an acid Yersinia mollaretii phytase for broadened activity at neutral pH. *Appl. Microbiol. Biotechnol.* **102**, 9607-9620, doi:10.1007/s00253-018-9308-7 (2018).

4 Rangou, S. *et al.* Self-organized isoporous membranes with tailored pore sizes. *Journal of Membrane Science* **451**, 266-275, doi:https://doi.org/10.1016/j.memsci.2013.10.015 (2014).

5 Stejskal, E. O. & Tanner, J. E. Spin diffusion measurements: spin echoes in the presence of a time-dependent field gradient. *The journal of chemical physics* **42**, 288-292 (1965).

6 Fierro, D., Scharnagl, N., Emmler, T., Boschetti-de-Fierro, A. & Abetz, V. Experimental determination of self-diffusivities through a polymer network for single components in a mixture. *Journal of membrane science* **384**, 63-71 (2011).

7 Zhang, Z., Rahman, M. M., Bajer, B., Scharnagl, N. & Abetz, V. Highly selective isoporous block copolymer membranes with tunable polyelectrolyte brushes in soft nanochannels. *Journal of Membrane Science* **646**, 120266, doi:https://doi.org/10.1016/j.memsci.2022.120266 (2022).

8 McKenna, S. M. *et al.* The continuous oxidation of HMF to FDCA and the immobilisation and stabilisation of periplasmic aldehyde oxidase (PaoABC). *Green Chemistry* **19**, 4660-4665, doi:10.1039/C7GC01696D (2017).

9 Birmingham, W. R. *et al.* Toward scalable biocatalytic conversion of 5-hydroxymethylfurfural by galactose oxidase using coordinated reaction and enzyme engineering. *Nature Communications* **12**, 4946, doi:10.1038/s41467-021-25034-3 (2021).

10 Sun, L., Petrounia, I. P., Yagasaki, M., Bandara, G. & Arnold, F. H. Expression and stabilization of galactose oxidase in Escherichia coli by directed evolution. *Protein Engineering, Design and Selection* **14**, 699-704, doi:10.1093/protein/14.9.699 (2001).

11 Ortiz, C. *et al.* Novozym 435: the “perfect” lipase immobilized biocatalyst? *Catalysis Science & Technology* **9**, 2380-2420, doi:10.1039/C9CY00415G (2019).

12 Höck, H. Development of engineered lipases for enhanced surface binding and polymerization applications by directed evolution PhD thesis, RWTH Aachen University, (2020).

13 Rübsam, K., Weber, L., Jakob, F. & Schwaneberg, U. Directed evolution of polypropylene and polystyrene binding peptides. *Biotechnol. Bioeng.* **115**, 321-330, doi:https://doi.org/10.1002/bit.26481 (2018).

14 Zhang, Z. *et al.* Quaternization of a Polystyrene-block-poly(4-vinylpyridine) Isoporous Membrane: An Approach to Tune the Pore Size and the Charge Density. *Macromol. Rapid Commun.* **40**, 1800729, doi:10.1002/marc.201800729 (2019).

15 Lotfi, S., Fischer, K., Schulze, A. & Schäfer, A. I. Photocatalytic degradation of steroid hormone micropollutants by TiO2-coated polyethersulfone membranes in a continuous flow-through process. *Nat. Nanotechnol.* **17**, 417-423 (2022).

16 Regmi, C. *et al.* Comparison of photocatalytic membrane reactor types for the degradation of an organic molecule by TiO2-coated PES membrane. *Catalysts* **10**, 725 (2020).

17 Smuleac, V., Butterfield, D. & Bhattacharyya, D. Layer-by-layer-assembled microfiltration membranes for biomolecule immobilization and enzymatic catalysis. *Langmuir* **22**, 10118-10124 (2006).

18 Clodt, J. I. *et al.* Double Stimuli-Responsive Isoporous Membranes via Post-Modification of pH-Sensitive Self-Assembled Diblock Copolymer Membranes. *Adv. Funct. Mater.* **23**, 731-738, doi:10.1002/adfm.201202015 (2013).

19 Tripathi, B. P., Dubey, N. C., Choudhury, S., Simon, F. & Stamm, M. Antifouling and antibiofouling pH responsive block copolymer based membranes by selective surface modification. *J. Mater. Chem. B* **1**, 3397, doi:10.1039/c3tb20386g (2013).

20 Lawrence, K. *et al.* "Hydrothermal wrapping" with poly(4-vinylpyridine) introduces functionality: pH-sensitive core–shell carbon nanomaterials. *J. Mater. Chem. A* **1**, 4559-4564, doi:10.1039/C3TA10198C (2013).

21 Yao, C., Li, X., Neoh, K. G., Shi, Z. & Kang, E. T. Surface modification and antibacterial activity of electrospun polyurethane fibrous membranes with quaternary ammonium moieties. *Journal of Membrane Science* **320**, 259-267, doi:https://doi.org/10.1016/j.memsci.2008.04.012 (2008).

22 Shi, Z., Neoh, K. G. & Kang, E. T. Antibacterial and Adsorption Characteristics of Activated Carbon Functionalized with Quaternary Ammonium Moieties. *Industrial & Engineering Chemistry Research* **46**, 439-445, doi:10.1021/ie0608096 (2007).

23 Zhang, Z. *et al.* Chemically Tailored Multifunctional Asymmetric Isoporous Triblock Terpolymer Membranes for Selective Transport. *Adv. Mater.* **32**, 1907014, doi:10.1002/adma.201907014 (2020).

24 Rouquerol, J. *et al.* Liquid intrusion and alternative methods for the characterization of macroporous materials (IUPAC Technical Report). *Pure Appl. Chem.* **84**, 107-136, doi:doi:10.1351/PAC-REP-10-11-19 (2011).

Uncropped scans of SDS-PAGE gels

For Supplementary Figure 1a:

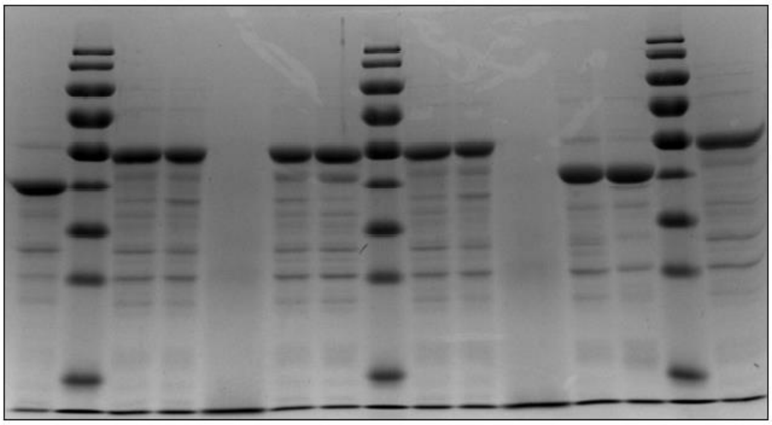

For Supplementary Figure 8:

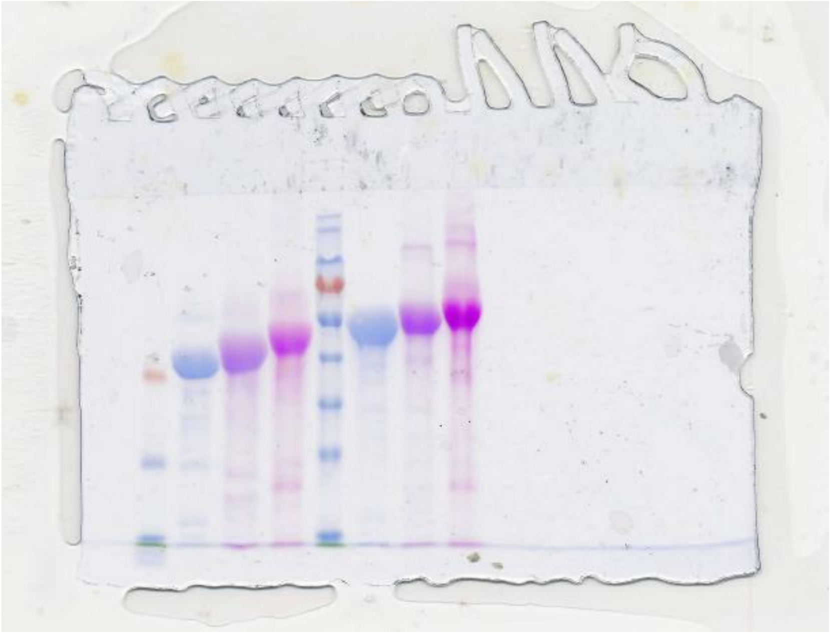

Supplement: Supplementary file 1 — Supplementary Information [file 41467_2024_47007_MOESM1_ESM.pdf]
